# Shade signals activate distinct molecular mechanisms that induce dormancy and inhibit flowering in vegetative axillary buds of sorghum

**DOI:** 10.1002/pld3.626

**Published:** 2024-08-19

**Authors:** Tesfamichael H. Kebrom

**Affiliations:** ^1^ Cooperative Agricultural Research Center, College of Agriculture, Food, and Natural Resources Prairie View A&M University Prairie View Texas USA; ^2^ Center for Computational Systems Biology, College of Engineering Prairie View A&M University Prairie View Texas USA

**Keywords:** axillary bud, defoliation, dormancy, shade signals, sorghum, *TERMINAL FLOWER*1

## Abstract

Shoot branches grow from axillary buds and play a crucial role in shaping shoot architecture and determining crop yield. Shade signals inactivate phytochrome B (phyB) and induce bud dormancy, thereby inhibiting shoot branching. Prior transcriptome profiling of axillary bud dormancy in a phyB‐deficient mutant (58M, *phyB‐1*) and bud outgrowth in wild‐type (100M, *PHYB*) sorghum genotypes identified differential expression of genes associated with flowering, plant hormones, and sugars, including *SbCN2*, *SbNCED3*, *SbCKX1*, *SbACO1*, *SbGA2ox1*, and *SbCwINVs*. This study examined the expression of these genes during bud dormancy induced by shade and defoliation in 100M sorghum. The aim was to elucidate the molecular mechanisms activated by shade in axillary buds by comparing them with those activated by defoliation. The expression of marker genes for sugar levels suggests shade and defoliation reduce the sugar supply to the buds and induce bud dormancy. Intriguingly, both shade signals and defoliation downregulated *SbNCED3*, suggesting that ABA might not play a role in promoting axillary bud dormancy in sorghum. Whereas the cytokinin (CK) degrading gene *SbCKX1* was upregulated solely by shade signals in the buds, the CK inducible genes *SbCGA1* and *SbCwINVs* were downregulated during both shade‐ and defoliation‐induced bud dormancy. This indicates a decrease in CK levels in the dormant buds. Shade signals dramatically upregulated *SbCN2*, an ortholog of the Arabidopsis *TFL1* known for inhibiting flowering, whereas defoliation did not increase *SbCN2* expression in the buds. Removing shade temporarily downregulated *SbCN2* in dormant buds, further indicating its expression is not always correlated with bud dormancy. Because shade signals also trigger a systemic early flowering signal, *SbCN2* might be activated to protect the buds from transitioning to flowering before growing into branches. In conclusion, this study demonstrates that shade signals activate two distinct molecular mechanisms in sorghum buds: one induces dormancy by reducing CK and sugars, whereas the other inhibits flowering by activating *SbCN2*. Given the agricultural significance of *TFL1*‐like genes, the rapid regulation of *SbCN2* by light signals in axillary buds revealed in this study warrants further investigation to explore its potential in crop improvement strategies.

## 1 INTRODUCTION

Plant shoot architecture is a major crop yield component, primarily determined by the number of shoot branches that develop from vegetative axillary buds (Sussex & Kerk, 2001). An axillary bud is an embryonic shoot with a few minute leaves that enclose and protect a meristem. The meristem in the bud possesses the same potential as the shoot apical meristem, including responding to systemic flowering signals generated in the main shoot. Interestingly, while the buds in maize and wheat transition directly to flowering, sorghum buds do not flower until they have grown into axillary shoots (Danilevskaya et al., 2010; Evers et al., 2006; Kebrom et al., 2012; Kebrom & Mullet, 2016). In annual plants, a newly formed bud enters dormancy if internal or environmental conditions are unfavorable for its sustained growth into a branch (Domagalska & Leyser, 2011; Luo et al., 2021; Shimizu‐Sato & Mori, 2001). Previous studies across various species identified internal factors, including plant hormones such as auxin, abscisic acid (ABA), cytokinin (CK), strigolactones, and sugars, which act within or outside the bud to induce dormancy or promote outgrowth (Barbier et al., 2019; Luo et al., 2021; Rameau et al., 2014; Wang et al., 2019). Although environmental factors such as limited water and nutrients can reduce overall plant growth, including shoot branching, mutual shading in densely planted areas promotes shoot elongation while inhibiting shoot branching (Casal, 2013; Kebrom, 2017; Schneider et al., 2019). Consequently, research on the environmental control of shoot branching has predominantly focused on elucidating how shade signals induce bud dormancy in plants.

Shade signals are detected by the light receptor phytochrome B (phyB), which is activated by red (R) light and deactivated by far‐red (FR) light (Martinez‐Garcia et al., 2010; Pierik & de Wit, 2014). The R:FR ratio of direct sunlight is about 1.2, driving a large fraction of phyB to the active state, but this ratio decreases and the fraction of active phyB is reduced at high planting density due to the absorption of R and reflection of FR by leaves (Ballare et al., 1990; Ballare & Pierik, 2017; Casal & Fankhauser, 2023). Under such conditions, plants initiate growth and developmental changes known as the shade avoidance syndrome (SAS), characterized by shoot elongation, early flowering, and reduced branching (Smith & Whitelam, 1997).

Research into the inhibition of shoot branching by shade signals has linked the activities of phyB with the expression of the *Teosinte branched1*‐like (*Tb1/BRC1*) genes in axillary buds (Aguilar‐Martinez et al., 2007; Finlayson et al., 2010; Kebrom et al., 2006). Mutants of *Tb1/BRC1* in diverse species including maize *tb1*, rice *fc1*, pea *Psbrc1*, and Arabidopsis *brc1* are highly branched (Aguilar‐Martinez et al., 2007; Braun et al., 2012; Finlayson, 2007; Hubbard et al., 2002; Takeda et al., 2003). The application of FR or a mutation in the *PHYB* gene, both of which deactivate phyB, increases the expression of the *Tb1/BRC1‐*like genes in the buds, inducing bud dormancy (Finlayson et al., 2010; Gonzalez‐Grandio et al., 2013; Kebrom et al., 2006). Conversely, high R:FR ratios activating phyB repress the *Tb1/BRC1* gene expression in axillary buds and promote bud outgrowth. However, the specific mechanisms through which the phyB‐Tb1/BRC1 pathway controls axillary bud dormancy and outgrowth remain unclear.

Transcriptome profiling of axillary bud dormancy in response to low R:FR ratios in Arabidopsis indicates that ABA promotes bud dormancy downstream of the phyB‐BRC1 pathway, evidenced by increased expression of the ABA biosynthesis gene *NCED3* and higher ABA levels in dormant buds (Gonzalez‐Grandio et al., 2017, 2013; Reddy et al., 2013). However, in sorghum, transcriptome studies revealed no significant difference in the expression of the sorghum ortholog of Arabidopsis *NCED3* (*SbNCED3*) between dormant buds of the phyB‐deficient mutant (58 M, *phyB‐1*) and growing buds of the near‐isogenic wild‐type (100 M, *PHYB*) plants (Kebrom & Mullet, 2016). Additionally, genes involved in deactivating CK (*cytokinin oxidase/dehydrogenase*, *SbCKX1*) and gibberellic acid (GA) (*GA2oxidase*, *SbGA2ox1*) were upregulated, whereas ethylene biosynthesis gene encoding *1‐Aminocyclopropane‐1‐Carboxylic Acid Oxidase* (*SbACO1*) was downregulated in the dormant buds of 58M plants (Kebrom & Mullet, 2016). Although CK is known to induce bud outgrowth when applied directly (Pillay & Railton, 1983; Turnbull et al., 1997), and light signals increase CK levels in rose buds (Roman et al., 2016), data suggesting a role for GA or ethylene in axillary bud dormancy or outgrowth are not evident (Chatfield et al., 2000; Cline, 1991).

Additionally, several sugar metabolism and responsive genes, including *cell wall invertases* (*SbCwINV1* and *SbCwINV2*) and *Trehalose Phosphate Phosphatase* (*TPP*), were regulated differently in the dormant buds of phyB mutant 58M compared to 100M sorghum plants (Kebrom & Mullet, 2016). For instance, *SbTPPI* expression was upregulated in the dormant buds of 58M sorghum. Studies in pea and Arabidopsis have shown that trehalose‐6‐phosphate (Tre6P) promotes shoot branching, and the *TPP* genes that catalyze the conversion of Tre6P into trehalose suppress bud outgrowth (Fichtner et al., 2021, 2017). The expression of the sorghum *SbCN2*, an ortholog of the Arabidopsis *TERMINAL FLOWER1* (*TFL1*) that inhibits the transition of vegetative meristems to flowering, was upregulated in the dormant buds of 58M plants (Kebrom & Mullet, 2016). While *TFL1* has been shown to induce bud dormancy in the perennial plant hybrid aspen (Maurya et al., 2020), the *SbCN2* in sorghum may inhibit premature transition of vegetative axillary buds to flowering (Kebrom & Mullet, 2016).

Previously, we identified that shade (supplemental FR light) and defoliation induce bud dormancy through partially distinct molecular mechanisms (Kebrom et al., 2010). The aim of this study was to gain a deeper understanding of the molecular mechanisms activated by shade signals in axillary buds by comparing them with those activated by defoliation. Therefore, in this study, the expression of the flowering, plant hormone, and sugar‐related genes identified in the transcriptome studies of 58M and 100M sorghum axillary buds—including *SbCN2*, *SbNCED3*, *SbGA2ox1*, *SbTTPI*, *SbCYP707A4*, *SbCKX1*, *SbACO1*, and *SbCwINVs*—was investigated in shade and defoliation‐induced bud dormancy in the wild‐type 100M sorghum plants. Currently, sugar supply from the parent shoot to the buds is the central research topic of axillary bud dormancy and outgrowth in annuals (Barbier et al., 2019; Kebrom, 2017; Kebrom & Doust, 2022; Schneider et al., 2019). The expression of marker genes for sugar levels was also investigated in the buds of 100M sorghum.

The results of this study delineate two groups of genes: those regulated by both shade signals and defoliation and those regulated solely by shade signals. Unexpectedly, the expression of *SbNCED3* was higher in growing buds and downregulated in the dormant buds of FR‐treated and defoliated plants, suggesting that ABA may not play a pivotal role in axillary bud dormancy in sorghum. Interestingly, shade signals, but not defoliation, upregulated *SbCN2* in dormant buds. Additionally, the expression of *SbCN2* is not always associated with bud dormancy. The study demonstrates that shade signals activate two distinct molecular mechanisms: one that induces bud dormancy and the other that inhibits flowering in vegetative axillary buds of sorghum, enhancing our knowledge of plant adaptive strategies under varying internal and environmental conditions.

## 2 RESULTS

### 2.1 The expression of sugar‐responsive genes in FR and defoliation‐induced bud dormancy

**FIGURE 1:**
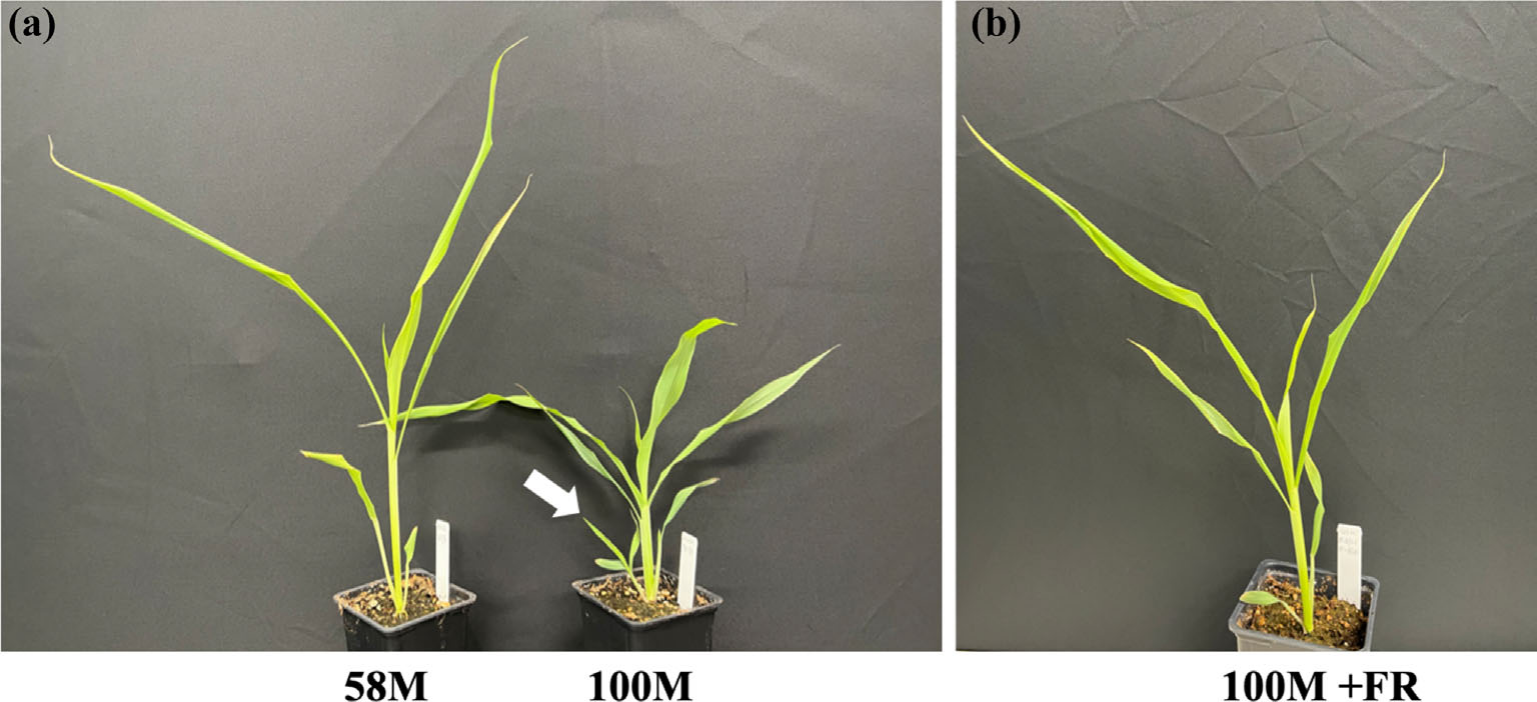
The response of sorghum plants to shade signals (supplemental far‐red light, FR) treatment. (a) A comparison between phyB‐deficient mutant (58M, *phyB‐1*) and near‐isogenic wild‐type (100M, *PHYB*) sorghum genotypes under growth conditions without FR. The arrow indicates the growth of a branch from the bud in the first leaf axil of 100M sorghum. (b) Effect of supplemental FR treatment (100M + FR) on shoot elongation and bud outgrowth inhibition in 100M sorghum plants. FR light treatment was initiated at 7 days after planting (DAP), with plant photographs taken at 12 DAP to visualize the impact of the additional FR on plant growth.

**FIGURE 2:**
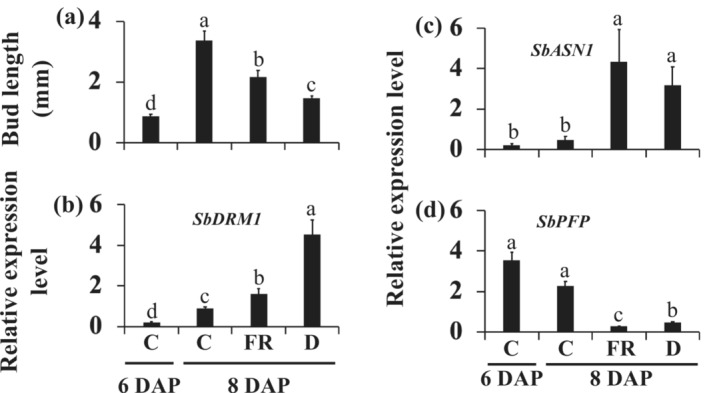
The response of buds in the first leaf axil of 100M sorghum plants to supplemental far‐red light (FR) and defoliation (D) treatments. (a) The effect of FR and defoliation initiated at 6 days after planting (6 DAP) on bud length. Data presented are means ± standard error of the means (SE) with *n* = 5. (b) Comparison of the expression levels of the dormancy associated *SbDRM1*, (c) sugar starvation‐inducible *SbASN1*, and (d) sugar‐inducible *SbPFP* genes in the buds of control (C) plants at 6 DAP and 8 DAP and in the FR‐treated and defoliated plants at 8 DAP. Expression data are means of three biological replicates ± standard error of the means (SE). Bars denoted by different letters are significantly different at *α* < .05.

Buds in the first leaf axil of the phyB‐deficient mutant 58M and its near‐isogenic wild‐type 100M sorghum genotypes are formed around 6 DAP. While the bud in 58M transitions into dormancy, the bud in 100M develops into a branch (Figure 1a). Previous studies have shown that shade signals (supplemental FR light) and defoliation applied around 6/7 DAP inhibit bud outgrowth in 100M plants (Kebrom et al., 2010, 2006); hence, we initially assessed the response of buds in 100M to supplemental FR, and defoliation treatments started at 6 DAP. As shown in Figure 1b, the height and shoot architecture of 100M grown with supplemental FR resemble the phyB‐deficient 58M. At 6 DAP, the average length of the bud in the first leaf axil in 100M control plants was .9 mm, increasing to 3.4 mm by 8 DAP (Figure 2a). However, in FR‐treated and defoliated 100M plants at 8 DAP, the average lengths of the buds were 2.2 and 1.5 mm, respectively. Additionally, the expression of the *dormancy‐associated* gene (*SbDRM1*) was higher in the buds of FR‐treated and defoliated plants (Figure 2b). The results indicate that FR and defoliation treatments initiated at 6 DAP inhibited bud outgrowth in 100M plants.

The expression of the Arabidopsis *glutamine‐dependent asparagine synthetase1* (*ASN1*) gene is upregulated by sugar starvation, whereas the expression of *pyrophosphate‐fructose‐6‐phosphate1‐phosphotransferase* (*PFP*) is upregulated by sugars (Gonzali et al., 2006). These genes serve as markers to characterize sugar levels in growing and dormant axillary buds. For example, bud growth arrest in the *tiller inhibition* (*tin*) mutant wheat (*Triticum aestivum*) correlated with the upregulation of *TaASN1*, downregulation of *TaPFP*, and reduced sugar levels in the buds (Kebrom et al., 2012). In this study, the inhibition of bud outgrowth by FR and defoliation was linked to the upregulation of the sorghum *SbASN1* and downregulation of *SbPFP1* genes (Figure 2c,d), indicating reduced sugar levels in the dormant buds.

### 2.2 The expression of plant hormone and flowering genes in FR and defoliation‐induced bud dormancy

**FIGURE 3:**
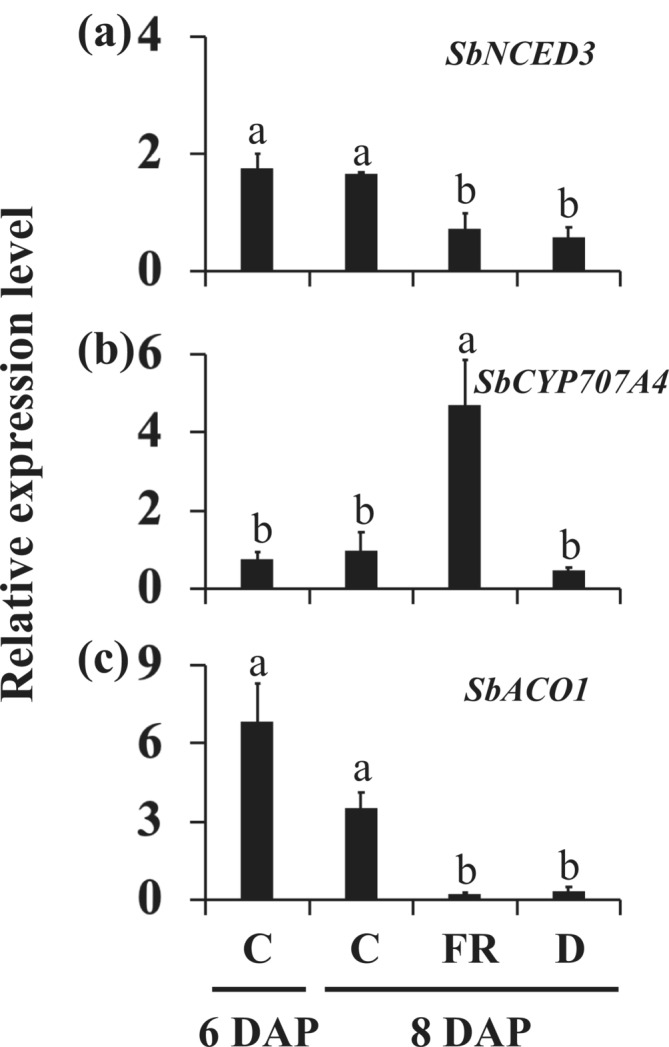
The expression of abscisic acid (ABA) and ethylene genes in growing and dormant buds of 100M sorghum plants subjected to supplemental far‐red light and defoliation treatments. (a) The expression of the ABA biosynthesis gene *SbNCED3*, (b) ABA deactivating gene *abscisic acid 8′hydroxylase* (*SbCYP707A4*), and (c) ethylene biosynthesis gene *ACC‐oxidase1* (*SbACO1*) in the growing buds of control (C) plants at 6 days after planting (DAP) and 8 DAP and dormant buds of plants treated with far‐red light (FR) and defoliated (D) at 8 DAP. Supplemental FR and defoliation treatments were started at 6 DAP. Data presented represent means of three biological replicates ± standard error of the means (SE). Bars denoted by different letters are significantly different at *α* < .05.

FR increased the expression of the ABA biosynthesis gene *NCED3* and the level of ABA in the Arabidopsis dormant buds (Gonzalez‐Grandio et al., 2013; Reddy et al., 2013). The expression level of *SbNCED3* remains unchanged from 6 DAP to 8 DAP in the growing buds of 100M control plants. In contrast, it decreased in the buds inhibited by FR and defoliation (Figure 3a). Furthermore, the expression of the *abscisic acid 8′‐hydroxylase* gene *SbCYP707A4* that degrades ABA was upregulated by FR in the buds (Figure 3b). The results suggest that the level of ABA was higher in growing than dormant buds of 100M sorghum plants.

While ethylene has not been associated with axillary bud dormancy or outgrowth in annual plants (Chatfield et al., 2000; Cline, 1991), transcriptome analyses of sorghum revealed the downregulation of the ethylene biosynthesis gene *ACC oxidase1* (*SbACO1*) in the dormant buds of 58M sorghum plants (Kebrom & Mullet, 2016). In this study, the expression of *SbACO1* was higher in growing control buds at 6 DAP and 8 DAP and downregulated in dormant buds of FR‐treated and defoliated plants (Figure 3c), indicating a growing bud in sorghum produces ethylene.

**FIGURE 4:**
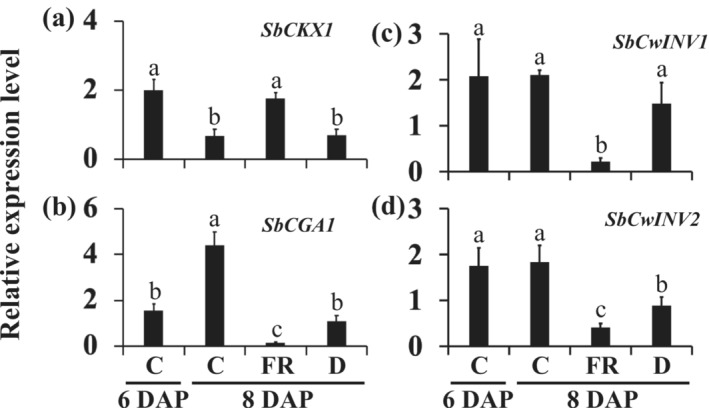
The expression of cytokinin (CK) and cell wall invertase (*SbCwINV*) genes in the buds of 100M sorghum plants subjected to supplemental far‐red light (FR) and defoliation treatments. (a) The expression levels of the cytokinin‐deactivating *SbCKX1*, (b) cytokinin‐inducible *SbCGA1*, and (c and d) *SbCwINV* genes in the growing buds of control (C) plants at 6 days after planting (DAP) and 8 DAP and dormant buds of plants treated with FR and defoliated (D) at 8 DAP. The supplemental FR light and defoliation treatments were initiated at 6 days after planting. The data presented are the means of three biological replicates ± standard error of the means (SE). Bars denoted by different letters are significantly different at *α* < .05.

Direct application of CK to dormant buds has been shown to stimulate bud outgrowth in various species (Cline, 1991; Pillay & Railton, 1983; Turnbull et al., 1997). The expression of the CK deactivating gene *SbCKX1* decreased from 6 DAP to 8 DAP in the growing buds of control and the dormant buds of defoliated 100M sorghum plants (Figure 4a). Additionally, the upregulation of the sorghum *CYTOKININ‐RESPONSIVE GATA TRANSCRIPTION FACTOR1* (*SbCGA1*) suggests an increase in CK levels in the growing buds of control plants at 8 DAP (Figure 4b), whereas its expression in the dormant buds of defoliated plants remained similar to that in the growing control buds at 6 DAP (Figure 4b). These results suggest that the reduction in *SbCKX1* expression did not increase CK levels in the buds of defoliated plants. Furthermore, the expression of *SbCKX1* in dormant buds of FR‐treated plants at 8 DAP did not decrease and was comparable with that in the control buds at 6 DAP (Figure 4a). The downregulation of *SbCGA1* indicates a higher level of *SbCKX1* expression maintained in the buds of plants treated with FR reduced CK levels in the buds (Figure 4b).

CK also promotes the expression of *CwINVs*, which encode enzymes that break down apoplastic sucrose into hexoses that can be imported by plant cells (Julius et al., 2017; Roitsch & Gonzalez, 2004). The expression of *SbCwINV2* decreased in the dormant buds of both FR‐treated and defoliated 100M sorghum plants (Figure 4d), whereas the expression of *SbCwINV1* was significantly reduced only in the buds of FR‐treated plants (Figure 4c). The expression level of *SbCwINV1* was about 7‐fold lower, and *SbCwINV2* was 2‐fold lower in the dormant buds of FR‐treated plants compared with those of defoliated plants. Expression trends of these genes followed similar patterns to that of *SbCGA1* under both FR and defoliation conditions.

**FIGURE 5:**
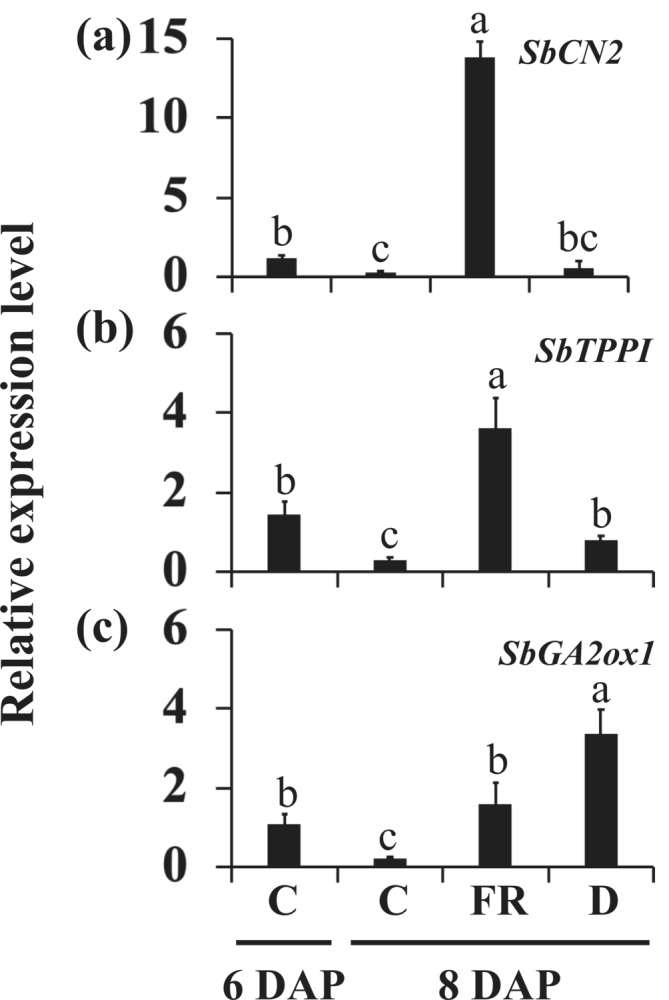
The expression of flowering‐related genes in the buds of 100M sorghum plants subjected to supplemental far‐red light (FR) and defoliation treatments. (a) The expression of the sorghum *TERMINAL FLOWER1 SbCN2*, (b) trehalose biosynthesis *SbTPPI*, and (c) gibberellic acid (GA) deactivating *SbGA2ox1* genes in the growing buds of control (C) plants at 6 days after planting (DAP) and 8 DAP and dormant buds of plants treated with FR and defoliated (D) at 8 DAP. Both supplemental FR light and defoliation treatments were initiated at 6 days after planting. The presented data are the means of three biological replicates ± standard error of the means (SE). Bars denoted by different letters are significantly different at *α* < .05.

Transcriptome studies of sorghum showed an increase in the expression of genes that inhibit flowering or inflorescence branching, such as the sorghum orthologs of the Arabidopsis *TFL1* (*SbCN2*), *TPPI* (*SbTPPI*), and *GA2ox1* (*SbGA2ox1*), in the dormant buds of phyB‐deficient 58M plants (Kebrom & Mullet, 2016). Because phytochrome deficiency also promotes early flowering of the shoot apical meristem, these genes were upregulated, possibly to inhibit the precocious transition of the vegetative axillary meristems to flowering until the buds grow into branches (Kebrom & Mullet, 2016). In this study, the expression of the *SbCN2*, *SbTPPI*, and *SbGA2ox1* genes in the growing control buds of 100M plants was reduced from 6 DAP to 8 DAP (Figure 5). The expression of *SbCN2* and *SbTPPI* was upregulated in the buds inhibited by FR but not by defoliation (Figure 5a,b). The expression of *SbGA2ox1* was significantly higher in the buds suppressed by FR and defoliation compared to the control at 8 DAP (Figure 5c).

### 2.3 Time course gene expression analysis in the buds of FR‐treated and defoliated plants

Defoliation induces bud dormancy earlier than supplemental FR, evidenced by the shorter buds at 8 DAP in defoliated 100M sorghum plants compared with those in FR‐treated plants (Figure 2a). To pinpoint the earliest physiological and molecular changes in the buds, we analyzed the expression of dormancy, sugar‐responsive, plant hormone, and flowering genes at 1, 3, and 6 h after supplemental FR and defoliation.

**FIGURE 6:**
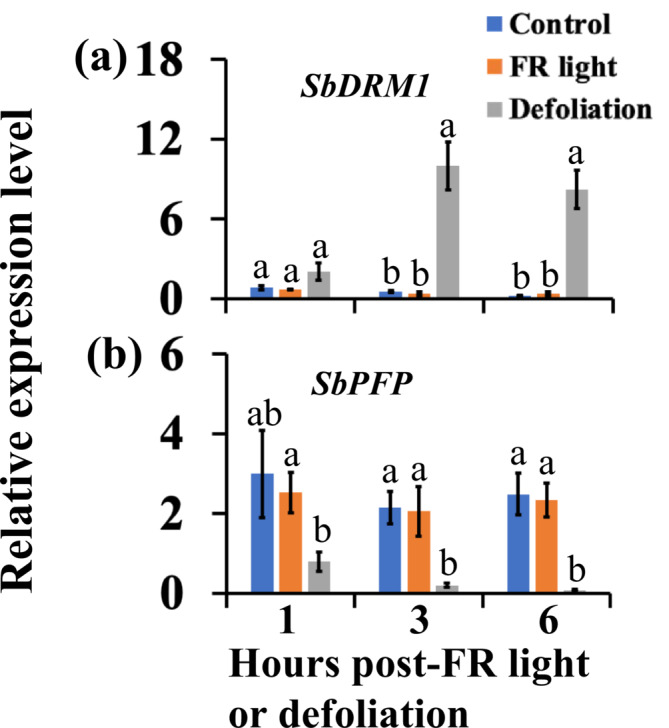
Time course gene expression analysis of the *dormancy‐associated SbDRM1* (a) and sugar‐inducible *SbPFP* (b) genes in buds of 100M sorghum plants. The analysis was conducted at 1, 3, and 6 h after supplemental far‐red light (FR) and defoliation treatments. The data presented represent the means of at least three biological replicates ± standard error of the means (SE). Significance testing was conducted at every time point, with bars marked by different letters indicating significant differences at *α* < .05.

The expression of *SbDRM1* slightly increased at 1 h after defoliation and further upregulated at 3 and 6 h after defoliation (Figure 6a). Conversely, the expression of the sugar‐inducible gene *SbPFP* was slightly reduced at 1 h and further reduced at 3 and 6 h after defoliation (Figure 6b). In contrast, during the first 6 h after FR, the expression of *SbDRM1* and *SbPFP* in the buds was not different than in the buds of untreated control plants (Figure 6a,b). The results suggest that defoliation immediately reduces the sugar supply to the buds and induces bud dormancy, unlike FR.

**FIGURE 7:**
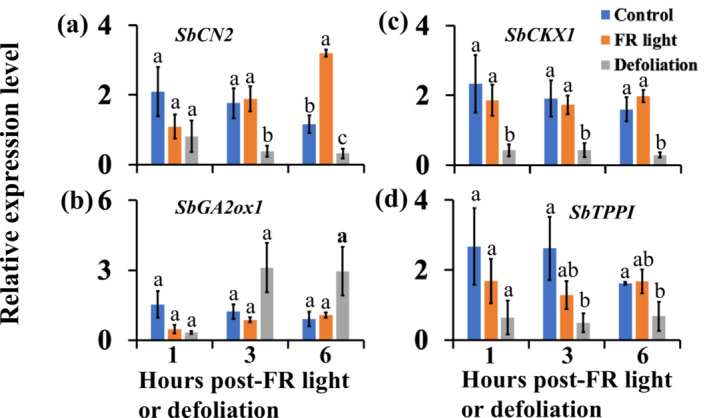
Time course gene expression analysis of the sorghum *TERMINAL FLOWER1* (*SbCN2*) (a), gibberellic acid deactivating *SbGA2ox1* (b), cytokinin deactivating *SbCKX1* (c), and trehalose biosynthesis *SbTPPI* (d) genes in buds of 100M sorghum plants. The analysis was conducted at 1, 3, and 6 h after supplemental far‐red light (FR) and defoliation treatments. The data presented are the means of a minimum of three biological replicates ± standard error of the means (SE). Significance testing was performed at every time point, with bars marked by different letters indicating significant differences at *α* < .05.

The expression of *SbCKX1* in the buds was reduced at 1 h after defoliation (Figure 7). At 3 and 6 h after defoliation, the expression of *SbCN2*, *SbCKX1*, and *SbTPPI* was reduced in the buds, whereas that of *SbGA2ox1* showed an increasing trend. Interestingly, *SbCN2* expression upregulated at 6 h after FR (Figure 7a). Meanwhile, the expression of *SbGA2ox1*, *SbTPPI*, and *SbCKX1* at 6 h after FR was not different from the control (Figure 7). These results from the time‐course study indicate that FR initiates physiological and molecular processes regulated by *SbCN2*, most likely inhibition of the flowering of the vegetative axillary buds, earlier than processes linked with bud dormancy, such as sugar level reduction in the buds.

### 2.4 The response of axillary buds to alternate‐day supplemental FR

**FIGURE 8:**
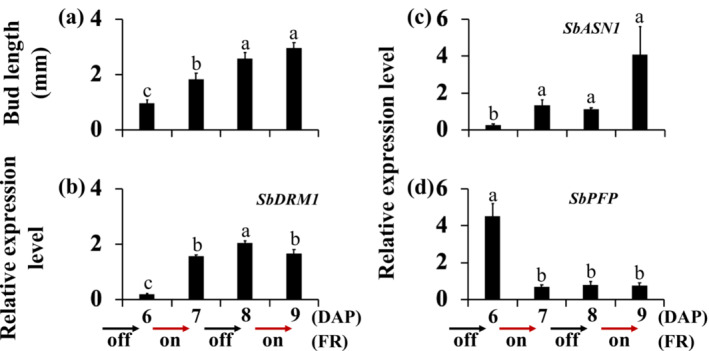
The response of sorghum buds to alternate‐day supplemental far‐red light (FR). 100M sorghum plants were grown without supplemental FR until 6 days after planting (DAP). The alternate‐day supplemental FR began at 6 h into the 14‐h light period at 6 DAP and persisted through the light period every other day (24 h) until 9 DAP. Bud length (a) and the expression of the dormancy‐associated *SbDRM1* (b), sugar starvation‐inducible *SbASN1* (c), and sugar‐inducible *SbPFP* (d) genes in the buds. Bud length data are means ± standard error of the means (SE); *n* = 5 buds. Gene expression data are the means obtained from three biological replicates ± standard error of the means (SE). Bars denoted by different letters are significantly different at *α* < .05.

While the expression of the sugar‐responsive genes suggests that both FR and defoliation reduced sugar supply to the buds and induced bud dormancy (Figure 2), FR additionally upregulates the expression of *SbCN2*, *SbTPPI*, *SbCKX1*, and *SbCYP707A4* genes in the dormant buds (Figures 3, 4, 5). The time course experiment revealed that only *SbCN2* was upregulated during the first 6 h after the initiation of FR treatment, suggesting rapid regulation of *SbCN2* by FR light. Because shade signals activate both flowering and dormancy‐associated genes, and *SbCN2* activation precedes dormancy genes, cycling FR light on and off every other day can provide further insights into gene responsiveness to shade signals. Consequently, 100M plants were subjected to alternate‐day supplemental FR commencing 6 h after the start of the 14 h light period at 6 DAP, continuing during the light period every other 24 h until 9 DAP. Sorghum being a short‐day plant, a 14 h light period was chosen to avoid photoperiodic induction of flowering. As shown in Figure 8a, the average bud length just before initiating supplemental FR at 6 DAP was 1.0 mm. By 7 DAP, after 24‐h FR treatment, the average bud length increased to 1.8 mm. Subsequently, the plants were grown without supplemental FR from 7 DAP, with an average bud length 2.6 mm by 8 DAP. Reintroducing supplemental FR at 8 DAP resulted in an average bud length of 3.0 mm by 9 DAP (Figure 8a). Notably, the bud length in the alternate‐day supplemental FR experiment at 8 DAP (2.6 mm) was shorter than that of the control plants (3.4 mm, Figure 2a) at the same time point. Thus, despite continuous bud growth, their overall growth rate was gradually diminished over the entire alternate‐day supplemental FR experimental period.

The expression of *SbDRM1* was low before the start of the alternate‐day supplemental FR at 6 DAP, increased to 8.2‐fold after 24 h FR treatment at 7 DAP, and remained elevated until 9 DAP (Figure 8b). The expression of the sugar starvation inducible *SbASN1* gene increased following the first 24 h supplemental FR from 6 DAP to 7 DAP and remained high at 8 DAP and 9 DAP (Figure 8c). Conversely, the expression of the sugar‐inducible gene *SbPFP* decreased following the initial 24‐h alternate‐day supplemental FR light from 6 DAP to 7 DAP and remained low until 9 DAP (Figure 8d). Collectively, the results suggest a reduction in the sugar supply from the parent shoot to the buds and the transition of the buds into dormancy throughout the entire period of alternated‐day supplemental FR, including at 8 DAP when the plants were not illuminated with supplemental FR during the preceding 24 h.

**FIGURE 9:**
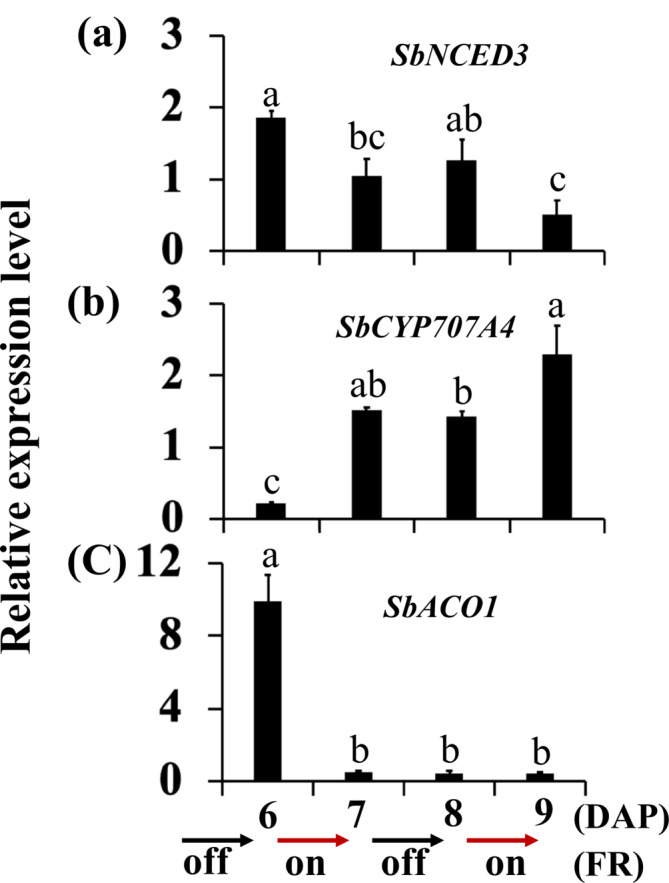
The expression of abscisic acid and ethylene genes in the sorghum buds under alternate‐day supplemental far‐red light (FR). 100M sorghum plants were initially grown without supplemental FR until 6 days after planting (DAP). Alternate‐day supplemental FR began at 6 h into the 14‐h light period at 6 DAP and continued every other day (24 h) until 9 DAP. The expression of ABA biosynthesis *SbNCED3* (a), ABA deactivating *abscisic acid 8′hydroxylase SbCYP707A4* (b), and ethylene biosynthesis *ACC‐oxidase1 SbACO1* (c) genes in the buds. The data represent the means from three biological replicates ± standard error of the means (SE). Bars denoted by different letters are significantly different at *α* < .05.

The expression of the ABA biosynthesis gene *SbNCED3* was reduced in the dormant buds of FR‐treated and defoliated plants (Figure 3a). Alternate‐day supplemental FR starting at 6 DAP reduced *SbNCED3* expression in the buds by 7 DAP, further decreasing when FR was reintroduced from 8 DAP to 9 DAP (Figure 9a). Temporarily removing supplemental FR from 7 DAP to 8 DAP slightly increased *SbNCED3* expression. Alternate‐day supplemental FR also upregulated the expression of *SbCYP707A4* that deactivates ABA (Figure 9b). The expression of the ethylene biosynthesis gene *SbACO1* in the buds was downregulated once the alternated‐day supplemental FR was started at 6 DAP, remaining low even when the FR was removed from 7 DAP to 8 DAP (Figure 9c). The results suggest a reduction in ABA and ethylene levels in the buds throughout the entire period of alternate‐day supplemental FR.

**FIGURE 10:**
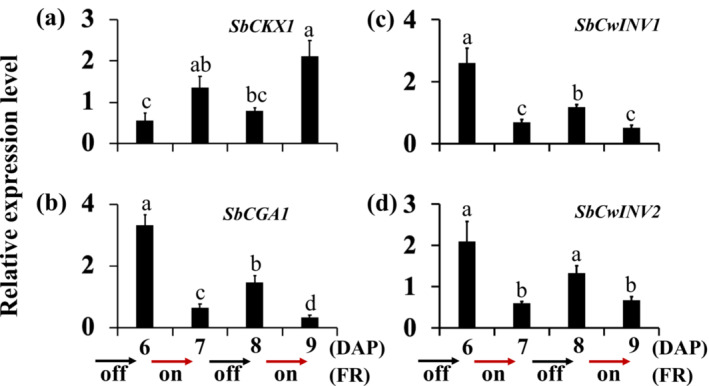
The expression of cytokinin and cell wall invertase (*SbCwINV*) genes in sorghum buds under alternate‐day supplemental far‐red light (FR). 100M sorghum plants were initially grown without supplemental FR until 6 days after planting (6 DAP). Subsequently, alternate‐day supplemental FR was introduced at 6 h into the 14‐h light period at 6 DAP and continued during the light period every other day (24 h) for the next 3 days. The expression of cytokinin‐deactivating *SbCKX1* (a), cytokinin‐responsive *SbCGA1* (b), *SbCwINV1* (c), and *SbCwINV2* (d) genes in the buds. The data presented are the means of three biological replicates ± standard error of the means (SE). Bars denoted by different letters are significantly different at *α* < .05.

Starting alternate‐day supplemental FR at 6 DAP increased the expression of the CK deactivating gene *SbCKX1* by 7 DAP, with subsequent reduction when FR was removed for the next 24 h by 8 DAP (Figure 10a). Reintroducing FR at 8 DAP increased *SbCKX1* expression by 9 DAP. Expression patterns of the CK‐responsive gene *SbCGA1* under alternate‐day supplemental FR were opposite to *SbCKX1*, being lower when *SbCKX1* expression was higher after 24‐h FR light at 7 DAP and 9 DAP and higher when *SbCKX1* expression was reduced when the 100M sorghum plants were not exposed to supplemental FR from 7 DAP to 8 DAP (Figure 10b). This contrasting expression suggests that FR increases *SbCKX1* expression, which, in turn, rapidly reduces the level of CK in the buds.

The expression of *SbCwINV1* and *SbCwINV2* was more reduced by FR than by defoliation (Figure 4c,d). Alternate‐day supplemental FR also altered the expression of the sorghum *SbCwINV* genes. *SbCwINV1* and *SbCwINV2* expression in buds of 100M sorghum plants decreased after 24‐h supplemental FR at 7 DAP and 9 DAP and increased when FR was removed from 7 DAP to 8 DAP (Figure 10c,d). The expression patterns of the *SbCwINV* genes aligned with the CK‐responsive gene *SbCGA1* (Figure 10b).

**FIGURE 11:**
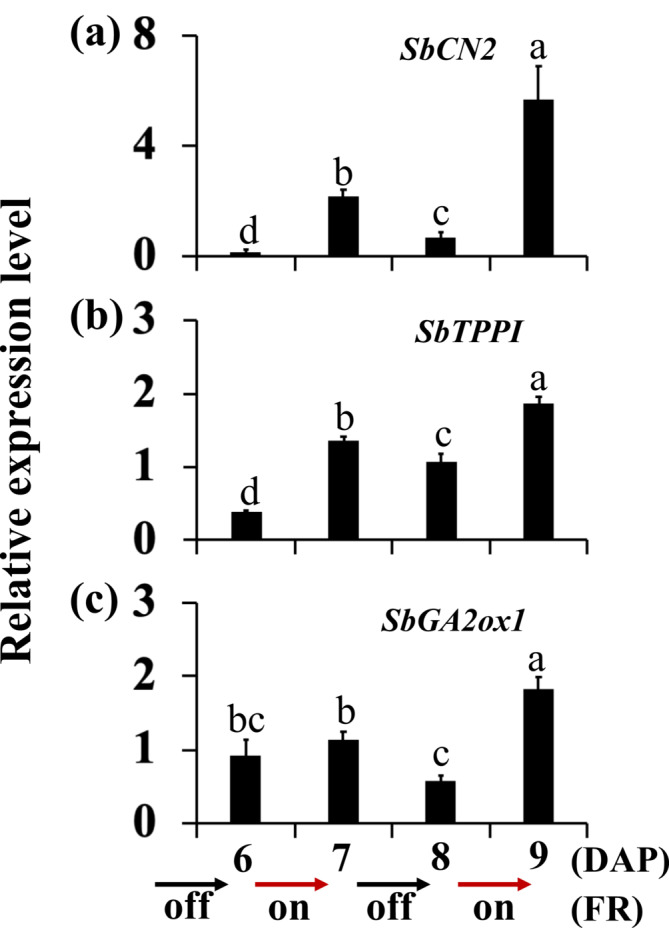
The expression of flowering‐related genes in buds of 100M sorghum plants under alternate‐day supplemental far‐red light (FR). The plants were initially grown without supplemental FR until 6 days after planting (6 DAP). Subsequently, alternate‐day supplemental FR was initiated at 6 h into the 14‐h light period at 6 DAP and continued during the light period every other day until 9 DAP. The expression of the sorghum *TERMINAL FLOWER1 SbCN2* (a), trehalose biosynthesis *SbTPPI* (b), and gibberellic acid (GA) deactivating *SbGA2ox1* (c) genes in the buds. The data presented represent the means of three biological replicates ± standard error of the means (SE). Bars denoted by different letters are significantly different at *α* < .05.

Initiating supplemental FR at 6 DAP upregulated *SbCN2* expression in the dormant buds of 100M plants by 8 DAP (Figure 5a). Alternate‐day supplemental FR experiments also showed that FR started at 6 DAP increased *SbCN2* expression by 7 DAP (Figure 11a). Interestingly, *SbCN2* expression in the buds downregulated when the plants were not illuminated with FR from 7 DAP to 8 DAP, despite *SbDRM1* upregulation indicating the dormancy status of the buds (Figure 8b). Reintroducing FR at 8 DAP, upregulated *SbCN2* expression by 9 DAP (Figure 11a). The extreme fluctuations of the *SbCN2* expression in response to alternate‐day supplemental FR demonstrate its rapid regulation by light signals in the sorghum buds, irrespective of bud dormancy status.

The expression of *SbTPPI* in 100M sorghum bud increased following the first 24‐h alternate‐day supplemental FR started at 6 DAP and following the second 24 h of FR started at 8 DAP (Figure 11b). Removing FR at 7 DAP slightly reduced *SbTPPI* expression by 8 DAP. The expression of *SbGA2ox1* decreased from 6 DAP to 8 DAP in the buds of 100M control plants (Figure 5c). Initiation of alternate‐day supplemental FR at 6 DAP prevented the decrease in *SbGA2ox1* expression in the buds until 7 DAP (Figure 11c). Removing FR at 7 DAP slightly reduced *SbGA2ox1* expression by 8 DAP compared to the level before starting FR at 6 DAP. Reintroducing supplemental FR at 8 DAP increased *SbGA2ox1* expression by 9 DAP, surpassing levels from the first FR treatment from 6 DAP to 7 DAP. Hence, the expression of *SbTPPI* and *SbGA2ox1* varied in the buds during the alternate‐day supplemental FR period.

## 3 DISCUSSION

Shade signals in sorghum promote shoot elongation, early flowering, and axillary bud dormancy (Kebrom & Mullet, 2016). However, the transition of meristems in dormant vegetative axillary buds into flowering does not occur simultaneously with the shoot apical meristem of the main and axillary shoots. Prior transcriptome profiling, conducted during the transition of sorghum axillary buds into dormancy in the phyB‐deficient mutant (58M, *phyB‐1*) and outgrowth in the near‐isogenic wild‐type (100M, *PHYB*) sorghum genotypes, revealed differential expression of genes that function in flowering as well as the synthesis or deactivation of plant hormones such as ABA, CK, GA, and ethylene (Kebrom & Mullet, 2016). Additionally, sugar‐related genes were differentially expressed in buds of 58M compared with those in 100M plants. Key genes identified in that study included *SbCN2*, *SbNCED3*, *SbGA2ox1*, *SbTTPI*, *SbCYP707A4*, *SbCKX1*, *SbACO1*, and *SbCwINVs*. In this study, aimed at gaining a deeper understanding of the molecular mechanisms activated by shade in axillary buds, their expression was analyzed in bud dormancy induced by shade signals (supplemental FR) and defoliation in 100M sorghum plants.

An upregulation of the sugar starvation‐inducible gene *SbASN1* and a downregulation of the sugar‐inducible gene *SbPFP* (Figure 2c,d) indicate that both FR and defoliation induce bud dormancy by diminishing the sugar supply to the buds. Several recent studies have also demonstrated decreased sugar levels in dormant axillary buds in diverse plant species (Bertheloot et al., 2020; Kebrom et al., 2012; Mason et al., 2014; Rabot et al., 2012; Schneider et al., 2019; Tarancon et al., 2017; Wang et al., 2021). Notably, defoliation altered the expression of marker genes for sugar level and induced bud dormancy earlier than FR. This outcome is anticipated because defoliation immediately halts sugar production in the shoot, and FR promotes shoot elongation—a stronger sink for sugars—and indirectly and gradually reduces the sugar supply to the buds (Kebrom, 2017).

Prior research has demonstrated higher levels of ABA in dormant buds compared with growing buds (reviewed in Cline, 1991; Shimizu‐Sato & Mori, 2001). Studies in Arabidopsis have also shown that FR inhibits bud outgrowth by upregulating the expression of the ABA biosynthesis gene *NCED3* and increasing ABA levels in the buds (Gonzalez‐Grandio et al., 2013; Reddy et al., 2013). In this study, the expression of *SbNCED3* was relatively high in growing buds and low in dormant buds of FR‐treated and defoliated 100M sorghum plants (Figure 3a). A lower level of ABA promotes seedling growth, and a higher level could deepen dormancy (Humplik et al., 2017). Hence, the relatively high expression levels of *SbNCED3* in the growing control buds suggest that ABA, possibly at a lower level, promotes bud outgrowth in sorghum. Furthermore, the expression of the *abscisic acid 8′‐hydroxylase* gene *SbCYP707A4* that degrades ABA was upregulated in the buds of FR‐treated plants (Figure 3b). Given that the R:FR ratio in a plant community fluctuates throughout the day, a decrease in *SbNCED3* and an increase in *SbCYP707A4* may prevent the buds from transitioning into deep dormancy in response to transient shade signals. Further investigation is warranted to elucidate the role of ABA in axillary bud dormancy and outgrowth in annual plants.

The *ACC oxidase* (*ACO*) gene encodes a rate‐limiting enzyme in ethylene biosynthesis (Houben & Van de Poel, 2019). In various species, a low R:FR ratio has been found to enhance *ACO* expression and ethylene production, including in shoots of Arabidopsis, sorghum, and others (Finlayson et al., 1999; Pierik et al., 2011). However, despite this general trend, the sorghum *SbACO1* was found to be downregulated in the dormant buds of the phyB‐deficient mutant 58M sorghum plants (Kebrom & Mullet, 2016). Interestingly, in this study, we observed an upregulation of *SbACO1* in growing buds and a downregulation in dormant buds of both FR‐treated and defoliated 100M sorghum plants (Figure 3c). Conclusive evidence regarding the role of ethylene in bud dormancy or outgrowth is still lacking (Chatfield et al., 2000; Cline, 1991). The higher expression of *SbACO1* in growing sorghum buds suggests a possible role in ethylene production to counter the pressure exerted by the overlying leaf sheath, akin to the ethylene production in germinating seeds to overcome soil barrier (Zhong et al., 2014; Zhu & Benkova, 2016), or when seedlings are physically entrapped by submergence (Voesenek et al., 2006). Previous suggestions of bud growth restriction by physical entrapment between the stem and leaf sheath in the grasses further support this idea (Williams & Metcalf, 1975), with extreme cases such as the *tiller inhibition* (*tin*) mutant wheat, where the stiff stem and leaf sheath in fully elongated mature shoots crush the axillary buds (Kebrom et al., 2012). Therefore, the expression of *SbACO1* may not be directly linked to the dormancy or outgrowth fates of the buds in sorghum. Growing buds in eudicots lack barriers like the leaf sheaths present in grasses and thus may not produce ethylene.

Shade signals have been shown to increase the expression of GA biosynthesis genes and GA levels (Yang & Li, 2017). GA is known to promote flowering in diverse plant species, including grasses (King & Evans, 2003; Leijten et al., 2018). *GA2oxidases*, expressed around the base of the shoot apex, prevent GA synthesized in leaf primordia and young leaves from inducing precocious transition of the shoot apical meristem to inflorescence meristem (Bolduc & Hake, 2009; Sakamoto et al., 2001). The increased expression of *SbGA2ox1* by FR light and defoliation in the bud of 100M sorghum (Figure 5c) may protect the axillary meristems from GA‐induced transition to flowering. Although GA is not known to play a role in axillary bud dormancy or outgrowth (Cline, 1991), a recent study demonstrated that GA promotes the growth of axillary buds that are released from dormancy (Cao et al., 2023). Further investigation is required to confirm whether GA promotes bud outgrowth or flowering of axillary buds of sorghum and other species.

CK is the only plant hormone known to promote axillary bud outgrowth (Cline, 1991; Pillay & Railton, 1983; Turnbull et al., 1997). Shade signals induce the expression of the CK‐deactivating gene *CKX* and inhibit leaf growth in Arabidopsis (Carabelli et al., 2007). In this study, shade signals upregulated *SbCKX1* and downregulated the CK‐inducible gene *SbCGA1* in dormant buds of 100M sorghum plants (Figure 4). Conversely, the expression of *SbCKX1* was reduced, and the expression of *SbCGA1* was increased in the growing buds of control plants. Although the expression of *SbCKX1* was reduced in dormant buds of defoliated plants, the expression of *SbCGA1* did not increase. These results suggest reduced CK transport to the buds in defoliated plants, possibly due to limited photosynthesis essential for CK production in the shoot and roots. Therefore, axillary bud dormancy may be induced by locally reducing CK levels through increased expression of *SbCKX1* under FR or reduced CK supply to the buds under defoliation.

CK and sugars activate the expression of *CwINVs*, facilitating the transport of apoplastic sucrose into developing sink tissues (Roitsch et al., 2000; Roitsch & Gonzalez, 2004). The expression of *SbCwINV1* and *SbCwINV2* was reduced in FR‐induced bud dormancy, and the expression of *SbCwINV2* was reduced in defoliation‐induced bud dormancy, more so by FR than defoliation (Figure 4d). The expression patterns of the *SbCwINVs* under alternate‐day supplemental FR were similar to the CK‐responsive gene *SbCGA1*. The results support the hypothesis that CK induces bud outgrowth by activating the expression of *CwINVs* and facilitating apoplastic sugar transport into axillary buds (Kebrom & Doust, 2022).

This study demonstrated that shade signals, but not defoliation, upregulated the sorghum *TFL1* gene *SbCN2* in the dormant axillary buds (Figure 5a). The *TFL1* gene in hybrid aspen promotes bud dormancy (Maurya et al., 2020), and *TFL1‐like* genes in annual plants delay the transition of vegetative meristems into flowering and maintain meristem indeterminacy (Ratcliffe et al., 1998; Zhu & Wagner, 2020). The vegetative meristems in axillary buds may perceive systemic flowering signals activated in the main shoot (Niwa et al., 2013). The shoot apical meristem and axillary meristems in some annual grasses, such as maize and wheat, synchronously switch to the flowering phase (Danilevskaya et al., 2010; Evers et al., 2006; Kebrom et al., 2012), whereas a sorghum plant can have axillary shoots at different stages of vegetative and reproductive development (Cox et al., 2018; Kebrom & Mullet, 2016). The upregulation of *SbCN2* in response to shade signals in sorghum buds could inhibit the flowering of vegetative axillary buds before growing into branches (Figure 5a). This hypothesis was reinforced by time course and alternate‐day supplemental FR light experiments (Figures 7a and 11a), demonstrating that the expression of *SbCN2* was rapidly upregulated in response to shade signals and downregulated upon removal of these signals, regardless of the dormancy or growth status of the buds. Interestingly, a mutation in the *TFL1* tomato gene *self‐pruning* promotes a synchronized transition of vegetative meristems to the flowering phase (Park et al., 2014). It would be intriguing to investigate if the synchronized flowering of vegetative shoot apical meristems and axillary buds in maize and wheat is due to a mutation in the *TFL1‐like* genes.

The *TPP* genes catalyze trehalose synthesis from Tre6P (Vandesteene et al., 2012). Over‐expression of *TPP* reduced shoot branching in Arabidopsis (Fichtner et al., 2021). The maize *RAMOSA3* (*RA3*) gene is an ortholog of the Arabidopsis *TPPI*, and the *ra3* mutant develops additional tassel and ear branches (Satoh‐Nagasawa et al., 2006). This study demonstrated the upregulation of *SbTPPI* by shade signals, and not defoliation, in dormant axillary buds (Figure 5b). Interestingly, the expression patterns of *SbTPPI* were similar to *SbCKX1*. In addition to bud outgrowth, CK promotes flowering and determinate growth in Arabidopsis by increasing the expression of the flowering genes *TWIN SISTER OF FT* (*TSF*) and *APETALA1* (D'Aloia et al., 2011; Han et al., 2014). Therefore, activation of *SbCKX1* may also protect the buds from CK‐induced flowering. However, additional research is required to ascertain whether *SbTPPI* and *SbCKX1* play a role in inhibiting the flowering of axillary buds in sorghum and other plant species.

## 4 CONCLUSION

**FIGURE 12:**
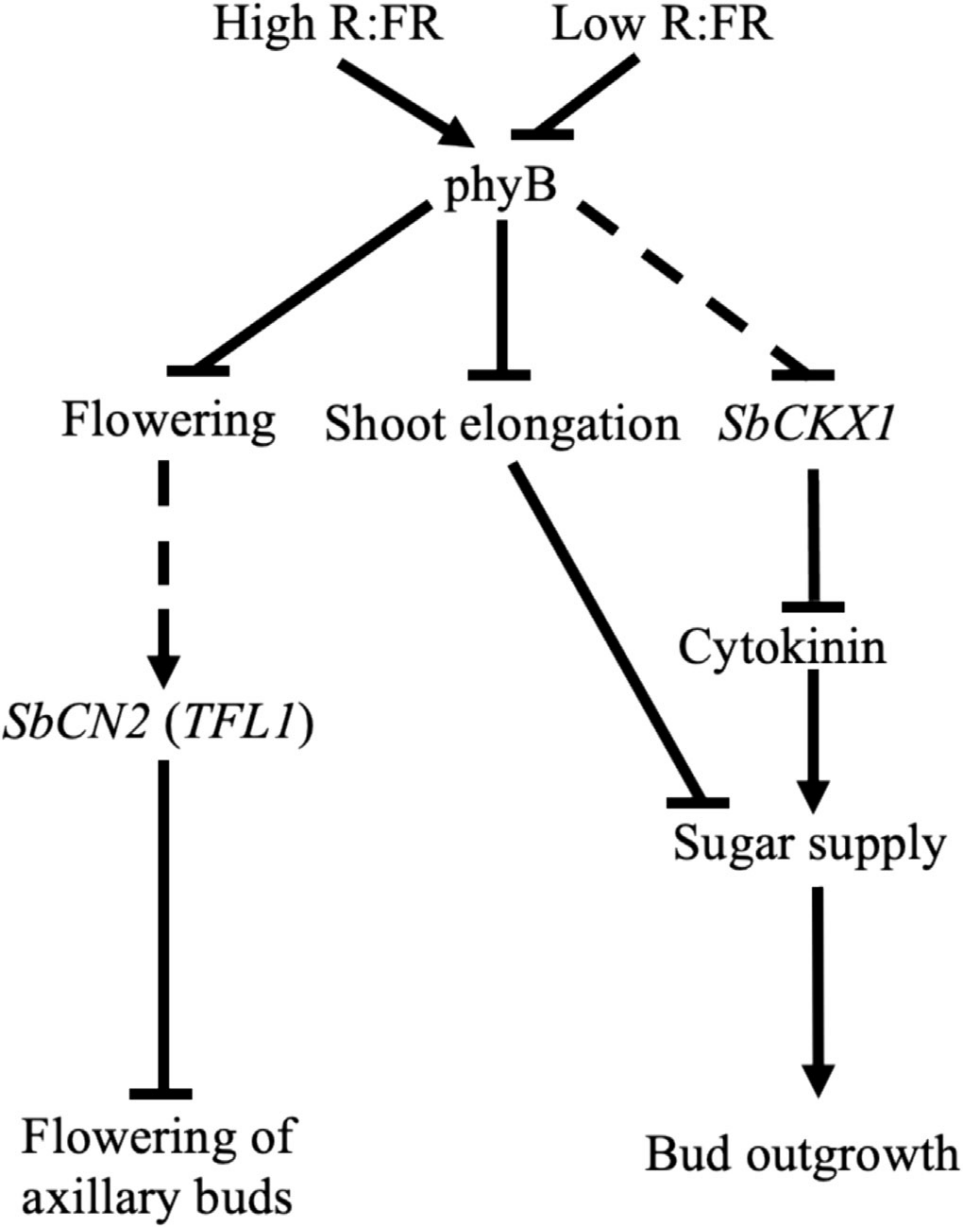
Model illustrating the molecular mechanisms inhibiting flowering and bud outgrowth in response to shade signal in sorghum. When shade signals (low red:far‐red [R:FR]) deactivate phytochrome B (phyB) in the leaves, this relieves flowering and shoot elongation signaling pathways imposed by phyB. Subsequently, the rapid upregulation of *SbCN2* inhibits the flowering of axillary buds. The increased sugar demand for elongating shoots diminishes the sugar supply to the buds, thereby inducing dormancy. Additionally, shade signals induce the upregulation of *SbCKX1* expression to counter cytokinin‐mediated sugar export into buds. The specific mechanisms through which shade signals trigger the activation of *SbCN2* and *SbCKX1* in axillary buds remain yet to be discovered, as indicated by the dashed lines denoting molecular mechanisms that are currently unknown.

Shade signals trigger various growth and developmental changes, such as increased shoot growth, early flowering, and decreased shoot branching. While prior research focused on how shade induces axillary bud dormancy and decreases shoot branching, this study reveals that shade also activates the sorghum gene *SbCN2*, an ortholog of the *TFL1* gene that inhibits flowering, in the buds. Consequently, a model shown in Figure 12 is proposed to illustrate the activation of distinct molecular processes that stimulate dormancy and inhibit flowering in axillary buds of sorghum. In this model, shade signals inhibit bud outgrowth primarily by reducing sugar availability. This reduction can occur either directly by stimulating shoot elongation, diverting sugars away from the buds, or indirectly by activating *SbCKX1*, which deactivates CK. This suggests that CK plays a role in facilitating sugar transport to the buds. Concurrently, activation of the sorghum *SbCN2* by shade signals inhibits the transition of the buds to the flowering phase. Therefore, this study highlights the need for future investigation into shade signaling and shoot branching to not only consider the dormancy and outgrowth of axillary buds but also the vegetative versus flowering fate of the buds. Additionally, *TFL1* is an agriculturally important gene. For instance, a mutation in the *SELF‐PRUNING* gene in tomato, which is an ortholog of the Arabidopsis *TFL1*, has facilitated synchronized flowering of meristems and enabled mechanized production of tomatoes (Park et al., 2014). Therefore, the discovery of light‐induced regulation of *SbCN2* in axillary buds in this study emphasizes the potential for similar advancements in crop production.

Unexpectedly, this study documented atypical expression patterns of plant hormone‐related genes in growing and dormant axillary buds. For example, even though ABA is recognized as a dormancy hormone, the ABA biosynthesis gene *SbNCED3* was downregulated in dormant sorghum buds. Moreover, the upregulation of the ethylene biosynthesis gene *SbACO1* in growing buds suggests ethylene production might aid their development into branches by overcoming physical barriers like the leaf sheath. These novel insights into the environmental and hormonal regulation of axillary bud dormancy and outgrowth highlight the importance of further research in understanding the complexities of shoot branching in annual plants.

## 5 MATERIALS AND METHODS

The materials and methods section of this study was previously detailed in works by Kebrom et al. (2010, 2006) and Kebrom and Mullet (2015, 2016), where the plant materials used were the phyB mutant 58M and near‐isogenic wild‐type 100M sorghum (*Sorghum bicolor*) genotypes. The current study focused on 100M sorghum plants. A concise overview of the materials and methods is described below.

### 5.1 Plant materials and growing conditions

100M sorghum plants were grown in a CONVIRON PGR15 plant growth chamber in trays containing 7‐cm deep cells filled with Miracle‐Gro commercial soil mix. Growth conditions include 30/22°C day/night temperatures, 14/10 h light/dark periods, and 50% relative humidity. Light in the chamber was provided by light‐emitting diodes (LEDs) with photosynthetically active radiation (PAR) of approximately 500 μMol m^−2^ s^−1^. Supplemental FR light was introduced as required during the 14 h light cycles.

### 5.2 FR and defoliation experiments

At 6 days after planting (6 DAP), the 100M sorghum plants developed two fully expanded leaves, with the emergence of the third leaf blade from the enclosing leaf sheath of the second leaf. The bud in the first leaf axil was observable under a dissecting microscope. Supplemental FR or defoliation was initiated at 6 DAP. The supplemental FR reduced the R:FR ratio to about .2, triggering a robust SAS response (Ballare & Pierik, 2017). Defoliation was applied by removing all the leaf blades, including the newly emerged third leaf. Because the bud has the potential to resume growth upon the emergence of new leaves from the defoliated plant (Kebrom & Mullet, 2015), newly developing leaf blades were continuously removed. Subsequently, at 8 DAP (48 h after FR or defoliation treatment), bud lengths in both control and treated samples were measured under a dissecting microscope. Buds sampled for gene expression analysis were placed in 10‐ to 20‐uL Lysis buffer (Thermo Fisher Scientific) and preserved in a −80°C freezer until RNA extraction.

For time course gene expression analysis, 100M plants were grown in the growth chamber until 6 DAP, and FR or defoliation was applied at 4 h after the start of the light period. Buds from the first leaf axil were sampled for RNA extraction at 1, 3, and 6 h after starting the FR or defoliation treatments. Buds were also harvested from untreated control plants at time intervals corresponding to when the buds from the FR‐treated and defoliated plants were sampled.

For alternate‐day supplemental FR experiments, 100M plants were grown in a plant growth chamber. The supplemental FR was started at 6 DAP by turning on FR light 6 h after the start of the 14‐h light period and was conducted every other 24 h until 9 DAP. Thus, the FR was turned on from 6 DAP to 7 DAP, turned off from 7 DAP to 8 DAP, and then turned on from 8 DAP to 9 DAP. A set of plants were sampled to determine bud length and to harvest buds for RNA extraction just before turning the FR on or off for the next 24 h, completing the sampling by 9 DAP.

### 5.3 RNA extraction, real‐time PCR (qPCR), and data analysis

RNA was extracted from buds using the TRIzol method (Invitrogen, Carlsbad, CA) and quantified using NanoDrop. About 1‐μg total RNA was treated with DNase I, and cDNA was prepared from half of the DNase I treated sample using the Superscript III cDNA preparation kit (Invitrogen). Real‐time PCR (qPCR) was performed in two technical replicates for each cDNA sample using a KICQSTART SYBR GREEN READYMIX (Sigma) on ViiA7 Real‐Time PCR System (Applied Biosystems). The remaining half of DNase I‐treated RNA corresponding to each sample was also assessed to check for DNA contamination of the RNA samples. Each sample was normalized using 18S rRNA and individually analyzed using the ∆∆CT method described in Kebrom et al. (2010). The relative expression levels shown represent the mean fold change derived from a minimum of three biological replicates. The qPCR primers used are provided in Table S1. Statistical analysis, involving comparisons between sample means of gene expression data and bud lengths, was carried out using Student's *t* test.

## AUTHOR CONTRIBUTIONS

THK designed and conducted the experiments, analyzed and interpreted the data, and wrote the paper.

## CONFLICT OF INTEREST STATEMENT

The author has no conflict of interest to declare.

## Supporting information


**Data S1.** Peer Review.

**Table S1:** Sorghum qPCR primers used in this study.

## Data Availability

The raw data are available from the author upon reasonable request.

## References

[pld3626-bib-0001] Aguilar‐Martinez, J. A. , Poza‐Carrion, C. , & Cubas, P. (2007). Arabidopsis BRANCHED1 acts as an integrator of branching signals within axillary buds. Plant Cell, 19, 458–472. 10.1105/tpc.106.048934 17307924 PMC1867329

[pld3626-bib-0002] Ballare, C. L. , & Pierik, R. (2017). The shade‐avoidance syndrome: Multiple signals and ecological consequences. Plant, Cell & Environment, 40, 2530–2543. 10.1111/pce.12914 28102548

[pld3626-bib-0003] Ballare, C. L. , Scopel, A. L. , & Sanchez, R. A. (1990). Far‐red radiation reflected from adjacent leaves: An early signal of competition in plant canopies. Science, 247, 329–332. 10.1126/science.247.4940.329 17735851

[pld3626-bib-0004] Barbier, F. F. , Dun, E. A. , Kerr, S. C. , Chabikwa, T. G. , & Beveridge, C. A. (2019). An update on the signals controlling shoot branching. Trends in Plant Science, 24, 220–236. 10.1016/j.tplants.2018.12.001 30797425

[pld3626-bib-0005] Bertheloot, J. , Barbier, F. , Boudon, F. , Perez‐Garcia, M. D. , Peron, T. , Citerne, S. , Dun, E. , Beveridge, C. , Godin, C. , & Sakr, S. (2020). Sugar availability suppresses the auxin‐induced strigolactone pathway to promote bud outgrowth. The New Phytologist, 225, 866–879. 10.1111/nph.16201 31529696

[pld3626-bib-0006] Bolduc, N. , & Hake, S. (2009). The maize transcription factor KNOTTED1 directly regulates the gibberellin catabolism gene GA2ox1. Plant Cell, 21, 1647–1658. 10.1105/tpc.109.068221 19567707 PMC2714931

[pld3626-bib-0007] Braun, N. , de Saint, G. A. , Pillot, J. P. , Boutet‐Mercey, S. , Dalmais, M. , Antoniadi, I. , Li, X. , Maia‐Grondard, A. , Le Signor, C. , Bouteiller, N. , Luo, D. , Bendahmane, A. , Turnbull, C. , & Rameau, C. (2012). The pea TCP transcription factor PsBRC1 acts downstream of Strigolactones to control shoot branching. Plant Physiology, 158, 225–238. 10.1104/pp.111.182725 22045922 PMC3252107

[pld3626-bib-0008] Cao, D. , Chabikwa, T. , Barbier, F. , Dun, E. A. , Fichtner, F. , Dong, L. , Kerr, S. C. , & Beveridge, C. A. (2023). Auxin‐independent effects of apical dominance induce changes in phytohormones correlated with bud outgrowth. Plant Physiology, 192, 1420–1434. 10.1093/plphys/kiad034 36690819 PMC10231355

[pld3626-bib-0009] Carabelli, M. , Possenti, M. , Sessa, G. , Ciolfi, A. , Sassi, M. , Morelli, G. , & Ruberti, I. (2007). Canopy shade causes a rapid and transient arrest in leaf development through auxin‐induced cytokinin oxidase activity. Genes & Development, 21, 1863–1868. 10.1101/gad.432607 17671088 PMC1935025

[pld3626-bib-0010] Casal, J. J. (2013). Photoreceptor signaling networks in plant responses to shade. Annual Review of Plant Biology, 64, 403–427. 10.1146/annurev-arplant-050312-120221 23373700

[pld3626-bib-0011] Casal, J. J. , & Fankhauser, C. (2023). Shade avoidance in the context of climate change. Plant Physiology, 191, 1475–1491. 10.1093/plphys/kiad004 36617439 PMC10022646

[pld3626-bib-0012] Chatfield, S. P. , Stirnberg, P. , Forde, B. G. , & Leyser, O. (2000). The hormonal regulation of axillary bud growth in Arabidopsis. The Plant Journal, 24, 159–169. 10.1046/j.1365-313x.2000.00862.x 11069691

[pld3626-bib-0013] Cline, M. G. (1991). Apical dominance. The Botanical Review, 57, 318–358. 10.1007/BF02858771

[pld3626-bib-0014] Cox, S. , Nabukalu, P. , Paterson, A. H. , Kong, W. , & Nakasagga, S. (2018). Development of perennial grain sorghum. Sustainability, 10, 172.

[pld3626-bib-0015] D'Aloia, M. , Bonhomme, D. , Bouche, F. , Tamseddak, K. , Ormenese, S. , Torti, S. , Coupland, G. , & Perilleux, C. (2011). Cytokinin promotes flowering of Arabidopsis via transcriptional activation of the FT paralogue TSF. The Plant Journal, 65, 972–979. 10.1111/j.1365-313X.2011.04482.x 21205031

[pld3626-bib-0016] Danilevskaya, O. N. , Meng, X. , & Ananiev, E. V. (2010). Concerted modification of flowering time and inflorescence architecture by ectopic expression of TFL1‐like genes in maize. Plant Physiology, 153, 238–251. 10.1104/pp.110.154211 20200067 PMC2862429

[pld3626-bib-0017] Domagalska, M. A. , & Leyser, O. (2011). Signal integration in the control of shoot branching. Nature Reviews. Molecular Cell Biology, 12, 211–221. 10.1038/nrm3088 21427763

[pld3626-bib-0018] Evers, J. B. , Vos, J. , Andrieu, B. , & Struik, P. C. (2006). Cessation of tillering in spring wheat in relation to light interception and red: Far‐red ratio. Annals of Botany, 97, 649–658. 10.1093/aob/mcl020 16464875 PMC2803660

[pld3626-bib-0020] Fichtner, F. , Barbier, F. F. , Annunziata, M. G. , Feil, R. , Olas, J. J. , Mueller‐Roeber, B. , Stitt, M. , Beveridge, C. A. , & Lunn, J. E. (2021). Regulation of shoot branching in arabidopsis by trehalose 6‐phosphate. The New Phytologist, 229, 2135–2151. 10.1111/nph.17006 33068448

[pld3626-bib-0021] Fichtner, F. , Barbier, F. F. , Feil, R. , Watanabe, M. , Annunziata, M. G. , Chabikwa, T. G. , Hofgen, R. , Stitt, M. , Beveridge, C. A. , & Lunn, J. E. (2017). Trehalose 6‐phosphate is involved in triggering axillary bud outgrowth in garden pea (*Pisum sativum* L.). The Plant Journal, 92, 611–623. 10.1111/tpj.13705 28869799

[pld3626-bib-0022] Finlayson, S. A. (2007). Arabidopsis teosinte Branched1‐like 1 regulates axillary bud outgrowth and is homologous to monocot teosinte Branched1. Plant & Cell Physiology, 48, 667–677. 10.1093/pcp/pcm044 17452340

[pld3626-bib-0023] Finlayson, S. A. , Krishnareddy, S. R. , Kebrom, T. H. , & Casal, J. J. (2010). Phytochrome regulation of branching in Arabidopsis. Plant Physiology, 152, 1914–1927. 10.1104/pp.109.148833 20154098 PMC2850038

[pld3626-bib-0024] Finlayson, S. A. , Lee, I. J. , Mullet, J. E. , & Morgan, P. W. (1999). The mechanism of rhythmic ethylene production in sorghum. The role of phytochrome B and simulated shading. Plant Physiology, 119, 1083–1089. 10.1104/pp.119.3.1083 10069847 PMC32090

[pld3626-bib-0025] Gonzalez‐Grandio, E. , Pajoro, A. , Franco‐Zorrilla, J. M. , Tarancon, C. , Immink, R. G. , & Cubas, P. (2017). Abscisic acid signaling is controlled by a BRANCHED1/HD‐ZIP I cascade in Arabidopsis axillary buds. Proceedings of the National Academy of Sciences of the United States of America, 114, E245–E254. 10.1073/pnas.1613199114 28028241 PMC5240681

[pld3626-bib-0026] Gonzalez‐Grandio, E. , Poza‐Carrion, C. , Sorzano, C. O. , & Cubas, P. (2013). BRANCHED1 promotes axillary bud dormancy in response to shade in Arabidopsis. Plant Cell, 25, 834–850. 10.1105/tpc.112.108480 23524661 PMC3634692

[pld3626-bib-0027] Gonzali, S. , Loreti, E. , Solfanelli, C. , Novi, G. , Alpi, A. , & Perata, P. (2006). Identification of sugar‐modulated genes and evidence for in vivo sugar sensing in Arabidopsis. Journal of Plant Research, 119, 115–123. 10.1007/s10265-005-0251-1 16463203

[pld3626-bib-0028] Han, Y. , Zhang, C. , Yang, H. , & Jiao, Y. (2014). Cytokinin pathway mediates APETALA1 function in the establishment of determinate floral meristems in Arabidopsis. Proceedings of the National Academy of Sciences of the United States of America, 111, 6840–6845. 10.1073/pnas.1318532111 24753595 PMC4020066

[pld3626-bib-0029] Houben, M. , & van de Poel, B. (2019). 1‐Aminocyclopropane‐1‐carboxylic acid oxidase (ACO): The enzyme that makes the plant hormone ethylene. Frontiers in Plant Science, 10, 695. 10.3389/fpls.2019.00695 31191592 PMC6549523

[pld3626-bib-0030] Hubbard, L. , McSteen, P. , Doebley, J. , & Hake, S. (2002). Expression patterns and mutant phenotype of teosinte branched1 correlate with growth suppression in maize and teosinte. Genetics, 162, 1927–1935. 10.1093/genetics/162.4.1927 12524360 PMC1462370

[pld3626-bib-0031] Humplik, J. F. , Bergougnoux, V. , & van Volkenburgh, E. (2017). To stimulate or inhibit? That is the question for the function of abscisic acid. Trends in Plant Science, 22, 830–841. 10.1016/j.tplants.2017.07.009 28843765

[pld3626-bib-0033] Julius, B. T. , Leach, K. A. , Tran, T. M. , Mertz, R. A. , & Braun, D. M. (2017). Sugar transporters in plants: New insights and discoveries. Plant & Cell Physiology, 58, 1442–1460. 10.1093/pcp/pcx090 28922744

[pld3626-bib-0034] Kebrom, T. H. (2017). A growing stem inhibits bud outgrowth—The overlooked theory of apical dominance. Frontiers in Plant Science, 8, 1874. 10.3389/fpls.2017.01874 29163599 PMC5671643

[pld3626-bib-0035] Kebrom, T. H. , Brutnell, T. P. , & Finlayson, S. A. (2010). Suppression of sorghum axillary bud outgrowth by shade, phyB and defoliation signalling pathways. Plant, Cell & Environment, 33, 48–58. 10.1111/j.1365-3040.2009.02050.x 19843258

[pld3626-bib-0036] Kebrom, T. H. , Burson, B. L. , & Finlayson, S. A. (2006). Phytochrome B represses teosinte Branched1 expression and induces sorghum axillary bud outgrowth in response to light signals. Plant Physiology, 140, 1109–1117. 10.1104/pp.105.074856 16443694 PMC1400571

[pld3626-bib-0037] Kebrom, T. H. , Chandler, P. M. , Swain, S. M. , King, R. W. , Richards, R. A. , & Spielmeyer, W. (2012). Inhibition of tiller bud outgrowth in the tin mutant of wheat is associated with precocious internode development. Plant Physiology, 160, 308–318. 10.1104/pp.112.197954 22791303 PMC3440208

[pld3626-bib-0038] Kebrom, T. H. , & Doust, A. N. (2022). Activation of apoplastic sugar at the transition stage may be essential for axillary bud outgrowth in the grasses. Frontiers in Plant Science, 13, 1023581. 10.3389/fpls.2022.1023581 36388483 PMC9643854

[pld3626-bib-0039] Kebrom, T. H. , & Mullet, J. E. (2015). Photosynthetic leaf area modulates tiller bud outgrowth in sorghum. Plant, Cell & Environment, 38, 1471–1478. 10.1111/pce.12500 25496467

[pld3626-bib-0040] Kebrom, T. H. , & Mullet, J. E. (2016). Transcriptome profiling of tiller buds provides new insights into PhyB regulation of tillering and indeterminate growth in sorghum. Plant Physiology, 170, 2232–2250. 10.1104/pp.16.00014 26893475 PMC4824614

[pld3626-bib-0041] King, R. W. , & Evans, L. T. (2003). Gibberellins and flowering of grasses and cereals: Prizing open the lid of the “florigen” black box. Annual Review of Plant Biology, 54, 307–328. 10.1146/annurev.arplant.54.031902.135029 14502993

[pld3626-bib-0042] Leijten, W. , Koes, R. , Roobeek, I. , & Frugis, G. (2018). Translating flowering time from *Arabidopsis thaliana* to brassicaceae and asteraceae crop species. Plants (Basel), 7, 111. 10.3390/plants7040111 30558374 PMC6313873

[pld3626-bib-0043] Luo, Z. , Janssen, B. J. , & Snowden, K. C. (2021). The molecular and genetic regulation of shoot branching. Plant Physiology, 187, 1033–1044. 10.1093/plphys/kiab071 33616657 PMC8566252

[pld3626-bib-0044] Martinez‐Garcia, J. F. , Galstyan, A. , Salla‐Martret, M. , Cifuentes‐Esquivel, N. , Gallemi, M. , & Bou‐Torrent, J. (2010). Regulatory components of shade avoidance syndrome. Advances in Botanical Research, 53, 65–116. 10.1016/S0065-2296(10)53003-9

[pld3626-bib-0045] Mason, M. G. , Ross, J. J. , Babst, B. A. , Wienclaw, B. N. , & Beveridge, C. A. (2014). Sugar demand, not auxin, is the initial regulator of apical dominance. Proceedings of the National Academy of Sciences of the United States of America, 111, 6092–6097. 10.1073/pnas.1322045111 24711430 PMC4000805

[pld3626-bib-0046] Maurya, J. P. , Miskolczi, P. C. , Mishra, S. , Singh, R. K. , & Bhalerao, R. P. (2020). A genetic framework for regulation and seasonal adaptation of shoot architecture in hybrid aspen. Proceedings of the National Academy of Sciences of the United States of America, 117, 11523–11530. 10.1073/pnas.2004705117 32393640 PMC7260942

[pld3626-bib-0047] Niwa, M. , Daimon, Y. , Kurotani, K. , Higo, A. , Pruneda‐Paz, J. L. , Breton, G. , Mitsuda, N. , Kay, S. A. , Ohme‐Takagi, M. , Endo, M. , & Araki, T. (2013). BRANCHED1 interacts with FLOWERING LOCUS T to repress the floral transition of the axillary meristems in Arabidopsis. Plant Cell, 25, 1228–1242. 10.1105/tpc.112.109090 23613197 PMC3663264

[pld3626-bib-0048] Park, S. J. , Jiang, K. , Tal, L. , Yichie, Y. , Gar, O. , Zamir, D. , Eshed, Y. , & Lippman, Z. B. (2014). Optimization of crop productivity in tomato using induced mutations in the florigen pathway. Nature Genetics, 46, 1337–1342. 10.1038/ng.3131 25362485

[pld3626-bib-0049] Pierik, R. , & de Wit, M. (2014). Shade avoidance: Phytochrome signalling and other aboveground neighbour detection cues. Journal of Experimental Botany, 65, 2815–2824. 10.1093/jxb/ert389 24323503

[pld3626-bib-0050] Pierik, R. , de Wit, M. , & Voesenek, L. A. (2011). Growth‐mediated stress escape: Convergence of signal transduction pathways activated upon exposure to two different environmental stresses. The New Phytologist, 189, 122–134. 10.1111/j.1469-8137.2010.03458.x 20854397

[pld3626-bib-0051] Pillay, I. , & Railton, I. D. (1983). Complete release of axillary buds from apical dominance in intact, light‐grown seedlings of *Pisum sativum* L. following a single application of cytokinin. Plant Physiology, 71, 972–974. 10.1104/pp.71.4.972 16662939 PMC1066154

[pld3626-bib-0052] Rabot, A. , Henry, C. , Ben Baaziz, K. , Mortreau, E. , Azri, W. , Lothier, J. , Hamama, L. , Boummaza, R. , Leduc, N. , Pelleschi‐Travier, S. , Le Gourrierec, J. , & Sakr, S. (2012). Insight into the role of sugars in bud burst under light in the rose. Plant & Cell Physiology, 53, 1068–1082. 10.1093/pcp/pcs051 22505690

[pld3626-bib-0053] Rameau, C. , Bertheloot, J. , Leduc, N. , Andrieu, B. , Foucher, F. , & Sakr, S. (2014). Multiple pathways regulate shoot branching. Frontiers in Plant Science, 5, 741. 25628627 10.3389/fpls.2014.00741 PMC4292231

[pld3626-bib-0054] Ratcliffe, O. J. , Amaya, I. , Vincent, C. A. , Rothstein, S. , Carpenter, R. , Coen, E. S. , & Bradley, D. J. (1998). A common mechanism controls the life cycle and architecture of plants. Development, 125, 1609–1615. 9521899 10.1242/dev.125.9.1609

[pld3626-bib-0055] Reddy, S. K. , Holalu, S. V. , Casal, J. J. , & Finlayson, S. A. (2013). Abscisic acid regulates axillary bud outgrowth responses to the ratio of red to far‐red light. Plant Physiology, 163, 1047–1058. 10.1104/pp.113.221895 23929720 PMC3793024

[pld3626-bib-0056] Roitsch, T. , Ehneß, R. , Goetz, M. , Hause, B. , Hofmann, M. , & Sinha, A. K. (2000). Regulation and function of extracellular invertase from higher plants in relation to assimilate partitioning, stress responses and sugar signalling. Australian Journal of Plant Physiology, 27, 815–825.

[pld3626-bib-0057] Roitsch, T. , & Gonzalez, M. C. (2004). Function and regulation of plant invertases: Sweet sensations. Trends in Plant Science, 9, 606–613. 15564128 10.1016/j.tplants.2004.10.009

[pld3626-bib-0058] Roman, H. , Girault, T. , Barbier, F. , Peron, T. , Brouard, N. , Pencik, A. , Novak, O. , Vian, A. , Sakr, S. , Lothier, J. , Le Gourrierec, J. , & Leduc, N. (2016). Cytokinins are initial targets of light in the control of bud outgrowth. Plant Physiology, 172, 489–509. 27462085 10.1104/pp.16.00530 PMC5074613

[pld3626-bib-0059] Sakamoto, T. , Kobayashi, M. , Itoh, H. , Tagiri, A. , Kayano, T. , Tanaka, H. , Iwahori, S. , & Matsuoka, M. (2001). Expression of a gibberellin 2‐oxidase gene around the shoot apex is related to phase transition in rice. Plant Physiology, 125, 1508–1516. 11244129 10.1104/pp.125.3.1508 PMC65628

[pld3626-bib-0060] Satoh‐Nagasawa, N. , Nagasawa, N. , Malcomber, S. , Sakai, H. , & Jackson, D. (2006). A trehalose metabolic enzyme controls inflorescence architecture in maize. Nature, 441, 227–230. 16688177 10.1038/nature04725

[pld3626-bib-0061] Schneider, A. , Godin, C. , Boudon, F. , Demotes‐Mainard, S. , Sakr, S. , & Bertheloot, J. (2019). Light regulation of axillary bud outgrowth along plant axes: An overview of the roles of sugars and hormones. Frontiers in Plant Science, 10, 1,296. 31681386 10.3389/fpls.2019.01296 PMC6813921

[pld3626-bib-0062] Shimizu‐Sato, S. , & Mori, H. (2001). Control of outgrowth and dormancy in axillary buds. Plant Physiology, 127, 1405–1413. 11743082 PMC1540171

[pld3626-bib-0063] Smith, H. , & Whitelam, G. C. (1997). The shade avoidance syndrome: Multiple responses mediated by multiple phytochromes. Plant, Cell and Environment, 20, 840–844.

[pld3626-bib-0064] Sussex, I. M. , & Kerk, N. M. (2001). The evolution of plant architecture. Current Opinion in Plant Biology, 4, 33–37. 11163165 10.1016/s1369-5266(00)00132-1

[pld3626-bib-0065] Takeda, T. , Suwa, Y. , Suzuki, M. , Kitano, H. , Ueguchi‐Tanaka, M. , Ashikari, M. , Matsuoka, M. , & Ueguchi, C. (2003). The OsTB1 gene negatively regulates lateral branching in rice. The Plant Journal, 33, 513–520. 12581309 10.1046/j.1365-313x.2003.01648.x

[pld3626-bib-0066] Tarancon, C. , Gonzalez‐Grandio, E. , Oliveros, J. C. , Nicolas, M. , & Cubas, P. (2017). A conserved carbon starvation response underlies bud dormancy in woody and herbaceous species. Frontiers in Plant Science, 8, 788. 28588590 10.3389/fpls.2017.00788 PMC5440562

[pld3626-bib-0067] Turnbull, C. G. N. , Raymond, M. A. A. , Dodd, I. C. , & Morris, S. E. (1997). Rapid increases in cytokinin concentration in lateral buds of chickpea (*Cicer arietinum* L.) during release of apical dominance. Planta, 202, 271–276.

[pld3626-bib-0068] Vandesteene, L. , Lopez‐Galvis, L. , Vanneste, K. , Feil, R. , Maere, S. , Lammens, W. , Rolland, F. , Lunn, J. E. , Avonce, N. , Beeckman, T. , & van Dijck, P. (2012). Expansive evolution of the trehalose‐6‐phosphate phosphatase gene family in Arabidopsis. Plant Physiology, 160, 884–896. 22855938 10.1104/pp.112.201400 PMC3461562

[pld3626-bib-0069] Voesenek, L. A. , Colmer, T. D. , Pierik, R. , Millenaar, F. F. , & Peeters, A. J. (2006). How plants cope with complete submergence. The New Phytologist, 170, 213–226. 16608449 10.1111/j.1469-8137.2006.01692.x

[pld3626-bib-0070] Wang, M. , le Moigne, M. A. , Bertheloot, J. , Crespel, L. , Perez‐Garcia, M. D. , Oge, L. , Demotes‐Mainard, S. , Hamama, L. , Daviere, J. M. , & Sakr, S. (2019). BRANCHED1: A key hub of shoot branching. Frontiers in Plant Science, 10, 76. 30809235 10.3389/fpls.2019.00076 PMC6379311

[pld3626-bib-0071] Wang, M. , Perez‐Garcia, M. D. , Daviere, J. M. , Barbier, F. , Oge, L. , Gentilhomme, J. , Voisine, L. , Peron, T. , Launay‐Avon, A. , Clement, G. , Baumberger, N. , Balzergue, S. , Macherel, D. , Grappin, P. , Bertheloot, J. , Achard, P. , Hamama, L. , & Sakr, S. (2021). Outgrowth of the axillary bud in rose is controlled by sugar metabolism and signalling. Journal of Experimental Botany, 72, 3044–3060. 33543244 10.1093/jxb/erab046

[pld3626-bib-0072] Williams, R. F. , & Metcalf, R. A. (1975). Physical constraint and tiller growth in wheat. Australian Journal of Botany, 23, 213–223.

[pld3626-bib-0073] Yang, C. , & Li, L. (2017). Hormonal regulation in shade avoidance. Frontiers in Plant Science, 8, 1,527. 28928761 10.3389/fpls.2017.01527 PMC5591575

[pld3626-bib-0074] Zhong, S. , Shi, H. , Xue, C. , Wei, N. , Guo, H. , & Deng, X. W. (2014). Ethylene‐orchestrated circuitry coordinates a seedling's response to soil cover and etiolated growth. Proceedings of the National Academy of Sciences of the United States of America, 111, 3913–3920. 24599595 10.1073/pnas.1402491111 PMC3964075

[pld3626-bib-0075] Zhu, Q. , & Benkova, E. (2016). Seedlings' strategy to overcome a soil barrier. Trends in Plant Science, 21, 809–811. 27553704 10.1016/j.tplants.2016.08.003

[pld3626-bib-0077] Zhu, Y. , & Wagner, D. (2020). Plant inflorescence architecture: The formation, activity, and fate of axillary meristems. Cold Spring Harbor Perspectives in Biology, 12, a034652. 31308142 10.1101/cshperspect.a034652 PMC6942122

