# Supplementary material for: Shade signals activate distinct molecular mechanisms that induce dormancy and inhibit flowering in vegetative axillary buds of sorghum
Source: Plant Direct. 2024 Aug 19;8(8):e626. doi: 10.1002/pld3.626 (PMC11333302; doi:10.1002/pld3.626)
Supplement: Supplementary file 2 — Table S1: Sorghum qPCR primers used in this study. [file PLD3-8-e626-s002.docx]

| **Gene** | **Sorghum Gene ID** | **Forward/Reverse primers** |
| --- | --- | --- |
| *SbDRM1* | Sobic.001G191200 | CATGGGTGCCAACCTCTTC/ATCGATCTAACGGTGGTTGC |
| *SbASN1* | Sobic.001G406800 | CGAGAAGTGGGTCATGAGGA/CCAACACCGTCACTGAACTG |
| *SbPFP* | Sobic.004G261800 | ATATGCAAGGTGAACGCACA/GAAGCCTTACGTCCCATCAG |
| *SbNCED3* | Sobic.001G155300 | GGTGCTGGACAAGGAGAAGA/GTTCCAAAGGTGGAAGCAGA |
| *SbCYP707A4* | Sobic.002G225400 | TCTTCTCCTCCTAGCCACCA/AGACTGTCGTTCCGCTGAGT |
| *SbACO1* | Sobic.003G197200 | CACAGTGTCGGAACTTGGAA/CTGAGATCGATCACCGGAAT |
| *SbCN2* | Sobic.004G165100 | AGCCTCTCATTGTGGGGAAA/TGCGGAAGGGAAGAACTCAT |
| *SbTPPI* | Sobic.010G089600 | TCAGATGCGTCGATGAAAAG/CAAGACTTTCCTGCCCTCAG |
| *SbGA2ox1* | Sobic.009G230800 | ACGGCTGTCGGTGATCTACT/GGTCTTGTACATGGCGGTCT |
| *SbCKX1* | Sobic.003G421100 | ACCAGGAACCACCCTCTTCT/GGCTAGCTCCTCCTCGATTT |
| *SbCGA1* | Sobic.004G094100 | GGCCAAGAAGGAGAAGAGGT/AGCAGCGTGATCGACAACTA |
| *SbCwINV1* | Sobic.006G070298 | AAGGCGGTCACTCTCAAGAA/CTGTGCTGTTTGTATCCCGG |
| *SbCwINV2* | Sobic.006G070564 | GGTGGTTGGAATGGTTGCTT/TAGACGACATGAGAAGCCCC |

**Table S1**: Sorghum qPCR primers used in this study.
