# Supplementary material for: Shade signals activate distinct molecular mechanisms that induce dormancy and inhibit flowering in vegetative axillary buds of sorghum
Source: Plant Direct. 2024 Aug 19;8(8):e626. doi: 10.1002/pld3.626 (PMC11333302; doi:10.1002/pld3.626)
Supplement: Supplementary file 1 — Data S1. Peer Review. [file PLD3-8-e626-s001.pdf]

Plant Direct
Open Access

Shade signals activate distinct molecular mechanisms that induce dormancy and inhibit flowering in vegetative axillary buds of sorghum

Tesfamichael H. Kebrom

Decision Letter Round 1:

March 20, 2024

Dr. Tesfamichael H Kebrom
Prairie View A&M University
Cooperative Agricultural Research Center, College of Agriculture and Human Sciences
620 E.E. O'Banion Street
P.O. Box 519; MS 2008
Prairie View, Texas 77446-0519

RE: Shade signals activate distinct molecular mechanisms that induce dormancy and inhibit flowering in vegetative axillary buds of sorghum

Dear Dr. Kebrom:

Thank you for submitting to Plant Direct. All required reviews have been returned and we have now finished our evaluation of your manuscript. In light of the reviewers' and editor's comments, further revisions are needed before the paper can be accepted for publication in Plant Direct.

Please view the editors' and reviewers' comments below and use their suggestions as a guide while you work on your revision.

When uploading the revised version of this article, please be sure to include the following:

-A word document that contains your response to the reviewers. You should respond to each reviewer comment and note the changes made to the manuscript. If you do not agree with a reviewer's comment and choose not to make a suggested revision, please explain why. Please try to provide as complete an answer as possible to each reviewer's criticisms in the "response to reviewers" section.
-A tracked changes document
- A clean version of the latest version of the manuscript

Plant Direct
Open Access

To upload your revision, please click the link below.
https://plantdirect.msubmit.net/cgi-bin/main.plex?el=A2Lr5gd4A2mjn1I5A9ftdsRKAe2NeOhHC1oHqjsvDgZ

In order to provide as timely a service as possible, we ask that your revision is resubmitted within three months after receipt of this request. If an extension is needed, please send a request, along with a brief explanation, to the editorial office at plantdirect@wiley.com .

Thank you very much for giving us an opportunity to review your work. I look forward to receiving the next version.

Sincerely,
Hongtao Ji

--- Editor comments:

The article investigated the effect of shade on dormancy of axillary buds of sorghum. The overall organizational framework of the article needs to be further improved to make it more logical, including the abstract section should be concise, the results section needs to strengthen the linkage between the conclusions, and the presentation of the gene expression data in the results section needs to be more standardized, and it is better to add the results of the significant differences of the analysis of variance (ANOVA). The final discussion section needs to be more focused. In addition, the title needs to be revised to better fit the conclusions of the study.

--- Reviewer comments:
Reviewer #1:

SBCN2 is an ortholog of TFL1 which inhibits flowering. Expression studies were used to try and evaluate its role in response to shade and defoliation. The results show that both FR light and defoliation cause reduced branch growth. The gene expression data indicate that defoliation and FR treatment have different effects on gene expression, particularly CN2/TFL1.

It is very important for the authors to state the difference between artificial shade (reduced light intensity) verses shade caused by plant foliage - which also affects light quality. (68-71)

The flow of the abstract is unclear with several bullet type points being made without drawing the collective meaning clear. Perhaps as a consequence, the purpose of the research was unclear.
This kind of writing continued in many other parts of the manuscript such as 105-121. E.g., 175,6; again, poor flow from this sentence to the next. This needs to be addressed throughout the manuscript.

Relying on gene expression only to associate or disassociate ABA from bud outgrowth is highly problematic and conclusions need to be adjusted accordingly (e.g, line 144). The timing of the harvest of tissue for gene expression will also affect the results and interpretation and needs discussion.

437-8 the authors suggest that: "The results showing that the regulation of the sorghum TFL1 gene SbCN2 and dormancy genes by various treatments are not always correlated is an important advance" . Why is this the case?

A discussion is also needed about whether TFL1 is a committed step towards flowering. Also, discussion is needed on potential alternative hypotheses and how they could be tested.

There are few (any?) statistical tests given.

Minor comments:
Abstract: have these hormones have already been shown to have a role in bud outgrowth in sorghum?
32: stated: The results showed that shade signals and defoliation started at 6 days after planting. -: it is not clear what started: the treatment or the response to the treatment and if the later, what sort of response.

Line 70 and 71 the sentence is not so clear.
In line 80 to 81 meaning unclear and poorly connected later on with the next paragraph.
90-95: initially refers to diverse plants for branching in tb1/brc1 - has the PHYB regulation gene expression been shown for all the species mentioned - be explicit and give the references.
100- this evidence is not sufficient to say that ABA induces bud dormancy - add causal evidence or reword.
101- initial suggestion that NCED3 is not differentially expressed should have been followed up with a detailed analysis of whether other ABA related genes could be regulated to the same effect in sorghum.
119- be more specific about what sort of light.
121 GA story as given is incorrect - See Cao et al Plant Physiology from Beveridge lab.
Sentence 134 to 137 sentence is too long.
180-1 and 186-7 description is unclear.

Statement in line 188-189 has been repeated in introduction 118-119 with the same references in introduction and in line 381 and 382 in discussion. I think is not necessary to be mentioned three times.
Line 244-245 not so clearly explained why the regulation of these genes by shade signals in the bud should be investigated by growing them under alternate-day supplemental FR.
356 - refer to figure

Reviewer #2:

Before submitting a paper, you should proofread the grammar and format of the full text, especially the notes and references in this paper. Each experiment requires an independent control group, otherwise the reliability of the conclusions of the experimental group cannot be fully demonstrated.

Decision Letter Round 2:

June 15, 2024

Dr. Tesfamichael H Kebrom
Prairie View A&M University
Cooperative Agricultural Research Center, College of Agriculture and Human Sciences
620 E.E. O'Banion Street
P.O. Box 519; MS 2008
Prairie View, Texas 77446-0519

RE: Shade signals activate distinct molecular mechanisms that induce dormancy and inhibit flowering in vegetative axillary buds of sorghum

Dear Dr. Kebrom:

Thank you for submitting to Plant Direct. All required reviews have been returned and we have now finished our evaluation of your manuscript. In light of the reviewers' and editor's comments, further revisions are needed before the paper can be accepted for publication in Plant Direct.

Please view the editors' and reviewers' comments below and use their suggestions as a guide while you work on your revision.

When uploading the revised version of this article, please be sure to include the following:

-A word document that contains your response to the reviewers. You should respond to each reviewer comment and note the changes made to the manuscript. If you do not agree with a reviewer's comment and choose not to make a suggested revision, please explain why. Please try to provide as complete an answer as possible to each reviewer's criticisms.
-A tracked changes document with each change highlighted
- A clean version of the latest version of the manuscript

To upload your revision, please click the link below.
https://plantdirect.msubmit.net/cgi-bin/main.plex?el=A4Lr7gd2B6mjn5I3A9ftdsRKAe2NeOhHC1oHqjsvDgZ

In order to provide as timely a service as possible, we ask that your revision is resubmitted within three months after receipt of this request. If an extension is needed, please send a request, along with a brief explanation, to the editorial office at plantdirect@wiley.com .

Please note that, in addition to publishing reviewer comments, the author's responses to review comments will also be published alongside the final version of the paper. If you would not like the author's responses to be published, please contact the editorial office at plantdirect@wiley.com .

Thank you very much for giving us an opportunity to review your work. I look forward to receiving the next version.

Sincerely,

Hongtao Ji

Gustavo MacIntosh

Editor, Plant Direct

-- Editor comments:
The author should be commended for the new revised version, but there are still some problems.

Firstly, the abstract and conclusion sections of the paper have been improved from the previous draft. However, they still lack clarity and fail to highlight the key points. I strongly recommend that the author reorganises these sections to ensure logical and clear content, with a clear purpose.

Secondly, I suggest the author make each Figure a separate one, instead of putting them together and using the same vertical coordinate. I also suggest the author put a,b,c,d to the outside of Figure.

Thirdly, there are also some formatting issues that require immediate attention. For instance, line 85, 'phyB' should be italicised as it refers to a gene; line 97, 108 and so on, 'phyB mutant' should be italicised; line 102 contains two full stops at the end of the sentence, which must be removed. Finally, line 476 should be µL. The authors must check the whole paper and revise the similar issues carefully.

Lastly, please address the queries raised by the second reviewer.

-- Reviewer comments:
Reviewer #2:

The author has made numerous revisions to the article, but there are still some issues. My suggestions are as follows:
Suggestion 1:
For section 2.4 of the result, it is recommended to introduce a control group that grows under normal conditions (without supplemental FR) to enhance the credibility of the experimental results.
Suggestion 2:
There are inaccuracies in multiple picture descriptions.
Figure 4, shows that in the dormant buds of defoliated plants, only SbCwINV2 expression is downregulated, while there is no significant difference observed in SbCGA1 and SbCwINV2. (192-196, 385-387)
In Figure 7, it is shown that almost all genes did not exhibit significant changes after treatment for 1 hour. How did you conclude: "The expression of SbCN2, SbGA2ox1, SbCKX1, and SbTPPI in the buds was reduced at 1 h after supplemental FR and/or defoliation treatments (Fig. 7)"(221-222)
Suggestion 3:
The significance notations on the bar chart are chaotic: "a, b, c, d" should be labeled in descending or ascending order of numerical values, and consistency should be ensured throughout the paper.
Suggestion 4:
Add image references in the discussion section to support arguments and enhance content visualization.

Suggestion 5:
There are many font and grammar issues.
The font of genes, genotypes, and Latin species names should be italicized.
"phyB (26, 30, 85, 97), phyb (421), TFL1 (48), etc."
The capitalization of the first letter of each word in a literature title should be consistent.
"Function and regulation of plant invertases: sweet sensations."
"Cytokinins Are Initial Targets of Light in the Control of Bud Outgrowth."

Decision Letter Round 3:

July 16, 2024
Dr. Tesfamichael H Kebrom
Prairie View A&M University
Cooperative Agricultural Research Center, College of Agriculture and Human Sciences
620 E.E. O'Banion Street
P.O. Box 519; MS 2008
Prairie View, Texas 77446-0519

MSID: 2024-01448R2
MS TITLE: Shade signals activate distinct molecular mechanisms that induce dormancy and inhibit flowering in vegetative axillary buds of sorghum

Dear Dr. Tesfamichael Kebrom:

I am pleased to inform you that your manuscript "Shade signals activate distinct molecular mechanisms that induce dormancy and inhibit flowering in vegetative axillary buds of sorghum" has been accepted for publication in Plant Direct.

Your article will appear online in the next available issue of Plant Direct. To ensure your article gets published as quickly as possible, please pay attention to the steps detailed below. We have found that most of the delays happen at this stage, especially at the payment stage, so please respond as quickly as possible when prompted.

License Agreement: Once your article has been accepted it will move to Production and undergo admin and file checking - you may receive an email with any queries we have at this stage. When all required items are received by the publisher and queries resolved, the corresponding author will receive an email from Wiley's Author Services system which will ask them to log on at https://authorservices.wiley.com/bauthor and will present them with the appropriate license for completion. Your article cannot be published until both the signed license agreement and payment of the article fee have been received.

Payment of the Open Access Article Publication Fee: All articles published in Plant Direct are fully open access: immediately and freely available to read, download and share. Plant Direct charges a publication fee to cover the publication costs. The corresponding author for this manuscript should have already received a quote from the payments team (cs-openaccess@wiley.com) with the estimated article publication fee; please email cs-openaccess@wiley.com if this has not been received. The corresponding author should log on to the Wiley Author Services site, where the publication fee can be paid by credit card or an invoice. Pro Forma can also be requested. Payment of the publication charge must be received before the article will be published online.

Proofs: You will have the opportunity to look over your paper once more when you receive the author proofs for your article. The proofs will be with you in approximately two weeks. Please note that, in addition to publishing reviewer comments, the author's responses to review comments will also be published alongside the final version of the paper. If you would not like the author's responses to be published, please contact the editorial office at plantdirect@wiley.com .

Promotion of your article: You can help your research get the attention it deserves! Check out Wiley's free Promotion Guide for best-practice recommendations for promoting your work at www.wileyauthors.com/eeo/guide. And learn more about Wiley Editing Services which offers professional video, design, and writing services to create shareable video abstracts, infographics, conference posters, lay summaries, and research news stories for your research at www.wileyauthors.com/eeo/promotion.

Thank you again for your contribution to Plant Direct. If you have any questions, feel free to contact the editorial office at plantdirect@wiley.com .

Sincerely,

Hongtao Ji

Gustavo MacIntosh

Editor, Plant Direct

--- Editor comments

--- Reviewer comments:

Author’s Response round 1:

Response to reviewer comments:

Reviewer #1:

Reviewer #1: SBCN2 is an ortholog of TFL1 which inhibits flowering. Expression studies were used to try and evaluate its role in response to shade and defoliation. The results show that both FR light and defoliation cause reduced branch growth. The gene expression data indicate that defoliation and FR treatment have different effects on gene expression, particularly CN2/TFL1.

It is very important for the authors to state the difference between artificial shade (reduced light intensity) verses shade caused by plant foliage - which also affects light quality. (**68-71**)

Author’s response: I agree with the reviewer's assessment that in natural environments, a plant shaded by another above it typically experiences both diminished light intensity and a decrease in the ratio of red to far-red (R:FR) light. However, in agricultural settings characterized by monoculture cultivation, a plant can detect shade signals primarily through a lowered R:FR ratio, due to the reflection of far-red (FR) light from neighboring plants of similar height. Plants can perceive and respond to reflected light from neighbor plants prior to actual shading without encountering a reduction in overall light intensity. For further information on this phenomenon, refer to the study by Ballare et al. (1990), titled "Far-red radiation reflected from adjacent leaves: an early signal of competition in plant canopies," published in Science (247, 329-332).

A more targeted investigation into the impact of shade signals on shoot branching or other responses to shading involves maintaining a constant light intensity while manipulating the R:FR ratio. This experimental design was feasible in the current study employing a plant growth chamber equipped with controllable supplemental FR lighting. Thus, the experimental approach adopted in this study stands as optimal, as shade signals were applied while ensuring consistent levels of photosynthetically active radiation. Nonetheless, I agree with the reviewer's suggestion that exploring scenarios that replicate shading between plants, encompassing reductions in both light quality and intensity, deserves investigation.

Reviewer #1: The flow of the abstract is unclear with several bullet type points being made without drawing the collective meaning clear. Perhaps as a consequence, the purpose of the research was unclear. This kind of writing continued in many other parts of the manuscript such as 105-121. E.g., 175,6; again, poor flow from this sentence to the next. This needs to be addressed throughout the manuscript.

Author's response: The abstract has been revised in response to feedback from the reviewer and editor. The updated version clearly articulates the research objectives and highlights the outcomes of the study.

I agree with the reviewer's comments regarding the redundancy and poor flow of statements in lines 105-121. Consequently, those statements have been removed in the updated version. Additional editing and improvement of the introduction section has been made, too.

Reviewer #1: Relying on gene expression only to associate or disassociate ABA from bud outgrowth is highly problematic and conclusions need to be adjusted accordingly (e.g, line 144). The timing of the harvest of tissue for gene expression will also affect the results and interpretation and needs discussion.

Author's response: The conclusion in Line 144: "The results suggest that ABA promotes bud outgrowth in sorghum" is replaced with "…ABA may not play a pivotal role in axillary bud dormancy in sorghum."

I agree with Reviewer #1 regarding the potential impact of harvest timing on the activation of clock-regulated genes. While the time-course experiments conducted in this study do not suggest the effect of harvest timing for the genes under analysis, I anticipate that the outcomes will stimulate additional research into the involvement of ABA in the dormancy of axillary buds in diverse plant species.

Reviewer #1: 437-8 the authors suggest that: "The results showing that the regulation of the sorghum TFL1 gene SbCN2 and dormancy genes by various treatments are not always correlated is an important advance" . Why is this the case?

Author's response: In our prior transcriptome investigations comparing the phyB mutant and wild-type sorghum (Kebrom and Mullet, 2016), we initially identified the activation of SbCN2 in dormant axillary buds. While it is well established that TFL1 suppresses flowering, the presence of SbCN2 in the dormant buds of phyB mutant sorghum could have dual effects, either inhibiting dormancy or flowering. However, our current study sheds light on this ambiguity by presenting evidence that SbCN2 doesn't consistently correlate with bud dormancy. Specifically, we found that SbCN2 expression occurs in dormancy induced by shade but not defoliation. Moreover, our time-course and alternate-day FR light experiments reveal that shade signals upregulate SbCN2 in growing buds while downregulating it in dormant buds. These findings underscore that SbCN2 expression isn't invariably linked with bud dormancy. This represents a significant advancement because, considering the insensitivity of sorghum buds to systemic flowering signals before growing into axillary shoots, our results suggest that SbCN2 isn't essential for dormancy, emphasizing its established role in suppressing flowering.

Reviewer #1: A discussion is also needed about whether TFL1 is a committed step towards flowering. Also, discussion is needed on potential alternative hypotheses and how they could be tested.

Author's response: TFL1 belongs to the same gene family as FT, a key promoter of flowering. TFL1 acts as an antagonist to FT, inhibiting the transition of meristems into the flowering phase, as noted in lines 121-125. While previous research on shoot branching has primarily concentrated on the dormancy and outgrowth of axillary buds, the present study highlights the activation of molecular mechanisms that determine the vegetative and flowering fates of the buds. I anticipate that the findings of this study will prompt investigations in other species, which could either corroborate the proposed hypothesis or lead to the development of alternative hypotheses.

Reviewer #1: There are few (any?) statistical tests given.

Author's response: I have addressed this concern by incorporating Student's t-test results directly into the figures. Additionally, I have added the following statements in the materials and methods: "Comparisons between sample means of bud length and gene expression data were conducted using Student's t-test."

Minor comments:
Reviewer #1: Abstract: have these hormones have already been shown to have a role in bud outgrowth in sorghum?
32: stated: The results showed that shade signals and defoliation started at 6 days after planting. -: it is not clear what started: the treatment or the response to the treatment and if the later, what sort of response.

Author's response: The roles of hormones in bud dormancy or outgrowth in sorghum have not been investigated, largely owing to the challenge of directly applying plant hormones to grass buds, enclosed with several layers of leaves and sheath, and directly observing responses. Nevertheless, the involvement of plant hormones in axillary bud dormancy and outgrowth appears to be conserved across various species. Furthermore, it is well known that the function of plant hormones in various aspects of plant growth and development, including environmental responses, is similar across diverse annual plant species, albeit with some variations in physiological or molecular mechanisms that await further investigations.

The statement in line 32: "The results showed that shade signals and defoliation **started** at 6 days after planting (6 DAP)…" Is now replaced with "The results showed that shade and defoliation **treatments initiated** at 6 DAP …."

Reviewer #1: Line 70 and 71 the sentence is not so clear.

Author's response: Line 70 to 71: "The regulation of branching by shade signals provides a relatively specific system to investigate how buds transition into and out of dormancy." is now replaced with "Hence, research on the environmental control of shoot branching has primarily focused on elucidating how shade signals induce axillary bud dormancy in plants."

Reviewer #1: In line 80 to 81 meaning unclear and poorly connected later on with the next paragraph.

Author's response: Line 81 to 84 is deleted in the updated version of the manuscript, I can see it really improved the flow. Thanks for this suggestion.

Reviewer #1: 90-95: initially refers to diverse plants for branching in tb1/brc1 - has the PHYB regulation gene expression been shown for all the species mentioned - be explicit and give the references.

Author's response: Yes, phyB regulation of tb1/BRC1 expression has been demonstrated in Arabidopsis and sorghum. The pioneering study by Kebrom et al. in 2006 was the first to show the regulation of TB1 by phyB/FR light in sorghum. Additionally, Finlayson et al. in 2010 further confirmed the regulation of BRC1 by phyB. However, certain studies, such as Aguilar-Martinez et al. in 2007, initially did not demonstrate the involvement of phyB or shade signals; instead, they focused on planting density. Hence, I cited their work as it was among the early demonstrations of environmental regulation of TB1. Nevertheless, in subsequent papers, they utilized FR light and showed the regulation of BRC1 by phyB. To adhere to precision, as recommended by the reviewer, Aguilar-Martinez et al., 2007 is replaced with Gonzalez-Grandio 2013.

Reviewer #1: Line 100- this evidence is not sufficient to say that ABA induces bud dormancy - add causal evidence or reword.

Author's response: Line 99-101: "The evidence includes increased expression of the ABA biosynthesis gene NCED3 and higher ABA levels in the dormant Arabidopsis buds." is now replaced with "The evidence **that suggests ABA induces bud dormancy** includes increased expression of the ABA biosynthesis gene NCED3 and higher ABA levels in the dormant Arabidopsis buds."

Reviewer #1: 101- initial suggestion that NCED3 is not differentially expressed should have been followed up with a detailed analysis of whether other ABA related genes could be regulated to the same effect in sorghum.

Author's response: The sorghum NCED3, an ortholog of Arabidopsis NCED3 identified in the buds, was discovered in our previous transcriptome study of sorghum axillary buds. Apart from NCED3, several other ABA-related genes were identified in the bud transcriptome, with their expression indicating a lower level of ABA in the buds. In this study, our focus was on key marker genes of each hormone, and NCED3 and the ABA deactivating gene were the two key genes for ABA included in this study.

Reviewer #1: 119- be more specific about what sort of light.

Author's response: In the study conducted by Roman et al., 2016, they utilized light white light versus dark conditions. Although the study wasn't specifically targeting shade signaling, it focused on the perception of light. Therefore, using "light" may be more suitable, as there is no mention of light of specific wavelengths in that study.

Reviewer #1: 121 GA story as given is incorrect - See Cao et al Plant Physiology from Beveridge lab.

Author's response: In Cao et al., two bud development stages are classified: bud release and the subsequent stage of bud growth. The study highlights a role for GA post bud release, indicating that GA does not influence bud release itself, the critical stage where dormancy versus outgrowth fates is determined. Consequently, their findings unequivocally establish GA's involvement after bud release, while suggesting a role for SL during the bud release stage. Thus, the current evidence still lacks support for a role of GA during the dormancy or outgrowth of axillary buds.

Reviewer #1: Sentence 134 to 137 sentence is too long.

Author's response: The sentence is now edited and reduced from 66 words to the following 38-word sentence: "Therefore, in this study, the expression of the flowering, plant hormone, and sugar-related genes, including SbCN2, SbNCED3, SbGA2ox1, SbTTPI, SbCYP707A4, SbCKX1, SbACO1 and SbCwINVs, was investigated in shade and defoliation-induced bud dormancy in the wild-type 100M sorghum plants."

Reviewer #1: 180-1 and 186-7 description is unclear.

Author's response: To make it clear the paragraph is now replaced with the following: "Ethylene hasn't been associated with axillary bud dormancy or outgrowth in annual plants (Chatfield et al., 2000; Cline, 1991). However, transcriptome analyses in sorghum revealed a downregulation of the ethylene biosynthesis gene ACC oxidase1 (SbACO1) in dormant buds of 58M sorghum (Kebrom and Mullet, 2016). In this study, SbACO1 expression was higher in actively growing control buds at 6 DAP and 8 DAP, but it was downregulated in dormant buds of plants treated with far-red light (FR) and defoliated plants (Fig. 3c), suggesting ethylene production by actively growing buds in sorghum."

Reviewer #1: Statement in line 188-189 has been repeated in introduction 118-119 with the same references in introduction and in line 381 and 382 in discussion. I think is not necessary to be mentioned three times.

Author's response: I agree that the statement has been reiterated multiple times in various sections of the manuscript. This is important to ensure that readers are reminded, as some may only focus on specific sections and could overlook key information pertaining to the data or discussion. The revised version now presents the statement in a different tone and context. Thank you for your suggestion.

Reviewer #1: 356 - refer to figure

Author's response: Figure is referred to. Thanks!

Reviewer #2:

Before submitting a paper, you should proofread the grammar and format of the full text, especially the notes and references in this paper. Each experiment requires an independent control group, otherwise the reliability of the conclusions of the experimental group cannot be fully demonstrated.

Author's response: Thank you for your valuable feedback. The manuscript has undergone a thorough review process and has been considerably enhanced. I trust that the revised version adequately addresses all of your concerns.

Author's Response round 2:

Plant Direct
Open Access

Editor comments:
The author should be commended for the new revised version, but there are still some problems.

Editor's comment: Firstly, the abstract and conclusion sections of the paper have been improved from the previous draft. However, they still lack clarity and fail to highlight the key points. I strongly recommend that the author reorganises these sections to ensure logical and clear content, with a clear purpose.

Author's response: The abstract and conclusion have been improved, clearly highlighting the study's purpose in the updated versions.

Editor's comment: Secondly, I suggest the author make each Figure a separate one, instead of putting them together and using the same vertical coordinate. I also suggest the author put a,b,c,d to the outside of Figure.

Author's response: The vertical coordinate for each figure is distinct. I believe readers will easily comprehend the figures without needing to refer to the text. The advice to place labels (a, b, c, d) outside of each figure is welcomed and implemented in the revised version, which enhanced the clarity of the figures.

Editor's comment: Thirdly, there are also some formatting issues that require immediate attention. For instance, line 85, 'phyB' should be italicised as it refers to a gene; line 97, 108 and so on, 'phyB mutant' should be italicised; line 102 contains two full stops at the end of the sentence, which must be removed. Finally, line 476 should be µL. The authors must check the whole paper and revise the similar issues carefully.

Author's response: All formatting issues, including those that were suggested for revision, have been thoroughly addressed in the revised manuscript.

Editor's comment: Lastly, please address the queries raised by the second reviewer.

-- Reviewer comments:
Reviewer #2:

The author has made numerous revisions to the article, but there are still some issues. My suggestions are as follows:

Reviewer's comment: Suggestion 1:
For section 2.4 of the result, it is recommended to introduce a control group that grows under normal conditions (without supplemental FR) to enhance the credibility of the experimental results.

Author's response: I appreciate the reviewer's feedback and understand their desire for more information. The bud length and expression of the genes under FR and in the phyB mutant compared to controls are well-established, making it unnecessary to repeat this analysis in the alternate-day FR experiment. For example, in section 2.4 (lines 248-250), it is mentioned that "*Notably, the bud length in the alternate-day supplemental FR experiment at 8 DAP (2.6 mm) was shorter than that of the control plants (3.4 mm, Fig. 2a) at the same time point.*" Under the alternate-day FR experiment, the gene expression level at 6 DAP before starting the alternate-day FR treatment serves as the control benchmark, with subsequent daily expressions with and without FR evaluated relative to this baseline and compared to prior days. Thus, monitoring gene expression patterns without FR (control) at 7 DAP, 8 DAP, and 9 DAP is redundant in this context.

Reviewer's comment: Suggestion 2:
There are inaccuracies in multiple picture descriptions.
Figure 4, shows that in the dormant buds of defoliated plants, only SbCwINV2 expression is downregulated, while there is no significant difference observed in SbCGA1 and SbCwINV2. (192-196, 385-387)
In Figure 7, it is shown that almost all genes did not exhibit significant changes after treatment for 1 hour. How did you conclude: "The expression of SbCN2, SbGA2ox1, SbCKX1, and SbTPPI in the buds was reduced at 1 h after supplemental FR and/or defoliation treatments (Fig. 7)"(221-222)

Author's response: Initially, the data was described as a trend rather than relying on statistically significant testing results. However, in the revised version, the presentation of results is now based on statistical testing outcomes, and necessary corrections, including those recommended by the reviewer, have been incorporated.

Reviewer's comment: Suggestion 3:
The significance notations on the bar chart are chaotic: "a, b, c, d" should be labeled in descending or ascending order of numerical values, and consistency should be ensured throughout the paper.

Author's response: I appreciate the suggestion and have made statistically significant annotations labeling based on descending order.

Reviewer's comment: Suggestion 4:
Add image references in the discussion section to support arguments and enhance content visualization.

Author's response: References to images have been included in the discussion. Thanks!

Reviewer's comment: Suggestion 5:
There are many font and grammar issues.
The font of genes, genotypes, and Latin species names should be italicized.
"phyB (26, 30, 85, 97), phyb (421), TFL1 (48), etc."
The capitalization of the first letter of each word in a literature title should be consistent.
"Function and regulation of plant invertases: sweet sensations."
"Cytokinins Are Initial Targets of Light in the Control of Bud Outgrowth."

Author's response: Font and grammar issues have been further corrected in the latest revised version. The inconsistencies in the references stem from using Endnote for generating the references. These issues are minor and corrected. Any additional issues related to formatting can be further corrected, if necessary, with the assistance of the editorial staff during proofreading,

I greatly appreciate the comments and suggestions of the editors and reviewers aimed at enhancing the manuscript!
